# Flavone-Based Anticancer Agents: Advances in Structure–Activity Relationships and Multitarget Mechanisms for Drug Design (2015–Present)

**DOI:** 10.3390/molecules31213866

**Published:** 2026-10-27

**Authors:** Mmakwena M. Mmonwa, Malose J. Mphahlele

**Affiliations:** 1 Department of Chemistry, KwaDlangezwa Campus, University of Zululand, Empangeni 3886, South Africa; 2 College of Science, Engineering and Technology, University of South Africa, Private Bag X06, Florida 1710, South Africa; mphahmj@unisa.ac.za

**Keywords:** flavones, flavonols, anticancer activity, biochemical targets, structure–activity relationship

## Abstract

2-Phenylchromen-4-one–based compounds (e.g., flavones and flavonols) have emerged as privileged scaffolds in anticancer drug discovery owing to their ability to modulate multiple molecular targets involved in tumour initiation, progression, metastasis, angiogenesis, immune evasion, and therapeutic resistance. This review correlates key structural modifications or the structure–activity relationships (SARs) of the naturally occurring and synthetic flavone analogues to enhance anticancer activity across diverse molecular targets from 2015 till present. The influence of hydroxylation, methoxylation, halogenation, sulphur bioisosteric replacement, heterocyclic substitution, and hybridisation strategies on target affinity, selectivity, pharmacokinetics, and biological efficacy is critically discussed. Emphasis is placed on flavone core interactions with various targets, highlighting representative lead compounds and their biological activities. Recent advances in computational modelling, molecular docking, and hybrid drug design further demonstrate the value of SAR-guided optimisation for improving potency and overcoming drug resistance. Collectively, the evidence establishes flavones as versatile multitarget anticancer scaffolds and provides a rational framework for developing next-generation flavone-based therapeutics with improved efficacy, selectivity, and drug-like properties.

## 1. Introduction

The 2-phenylchromen-4-one core represents a common basic skeleton for both flavones and flavonols. Flavones lack a hydroxyl residue present at position-3 of the C-ring of flavonols (hydroxyflavones). Flavones are not intermediates in the biosynthesis of flavonols and both classes of compounds are formed via a separate branch of the greater flavonoid biosynthetic network involving flavanones as common bio-precursors [1]. Flavone and its derivatives are present in large quantities in fruits and vegetables consumed by humans and other mammals in their daily diet and have been found to have a positive impact on human health with no detrimental side effects [2]. Numerous published reviews underpin the medicinal significance of flavones (2-arylchromones or 2-aryl-4*H*-chromen-4-ones) as the new chemical space in the design and development of small drug molecules against non-communicable diseases (NCDs), including cancer [3,4,5,6], cardiovascular diseases [7,8,9], diabetes [10,11] and neurodegenerative disorders [12,13]. More recent reviews have further highlighted flavones and other flavonoids as promising anticancer agents through their ability to disrupt multiple key molecular mechanisms that drive tumour development and spread [2,14], enhance tumour sensitivity to conventional therapies [15], and support precision medicine approaches. The significant role of C2–C3 double bond contributes to molecular planarity and conjugation between rings A/B and C, which is essential for potent tumour inhibition of apigenin (flavone) versus naringenin (flavanone), for example [16]. The therapeutic potential of the nature-based flavone derivatives is strongly influenced by their structural features, such as the number and position of hydroxyl [17], methoxy [1,18,19], and C- and/or *O*-glycosyl [20,21,22] substituents on the flavone scaffold. Extensive evidence indicates that these structural features determine interactions with key molecular targets involved in carcinogenesis, including the NF-κB (Nuclear Factor kappa-light-chain-enhancer of activated B cells) signalling pathway, which has emerged as an important target for flavonoid-based anticancer intervention [23]. Despite increased biological properties, the pharmaceutical application of the naturally occurring A-ring hydroxy-substituted flavone derivatives is limited by their reduced solubility and oral bioavailability due to the hydroxyl groups, which are also prone to rapid intestinal or hepatic conjugation via glucuronidation or sulfation [24,25]. Methylation of free hydroxyl groups (methoxylation) of the flavone scaffold, on the other hand, has been found to enhance membrane-penetrating properties leading to facilitated absorption and highly increased oral bioavailability [26,27]. 7-Hydroxyflavone, 7,4′-dihydroxyflavone, and 5,7-dihydroxyflavone (chrysin), for example, were undetectable in tissue levels after administration to rats, whereas the corresponding methylated derivatives reached high tissue levels [28]. The naturally occurring flavones such as luteolin and apigenin exemplify the influence of structural substitution on biological activity. Recent in vivo studies demonstrate potent anticancer efficacy alongside important considerations regarding toxicity and therapeutic windows [29]. Understanding SARs of nature-based and synthetic flavone analogues provides valuable insights into the molecular determinants responsible for their pharmacological effects, bioavailability, and target selectivity.

Several strategies for the derivatisation of the flavonoid core through the modifications of the flavonoid’s carbonyl group, hydroxyl group(s), phenyl, and pyran rings continue to be explored to enhance their drug-like properties and therapeutic potential and the methods for their transformation form part of several review articles [19,30,31,32]. For example, halogen-substituted flavones have demonstrated enhanced apoptotic activity and modulation of multiple cell death pathways in lymphoma and leukaemia models [33], whereas thioflavones have emerged as promising bioisosteric analogues with expanding synthetic methodologies and biological applications [34]. Most of the reviews published to-date, comprehensively discuss the general anticancer mechanisms of flavonoids and specific signalling pathways (e.g., NF-κB) of individual subclasses of flavones or selected structural analogues. Despite these encouraging developments, a comprehensive assessment that correlates structural modifications of both naturally occurring and synthetic flavones with their SARs across diverse cancer targets remains considerably less documented. Accordingly, this review focuses on the key structural modifications governing the anticancer activities of flavones and highlights their relevance in the prevention and management of cancer. By integrating medicinal chemistry, SAR analysis, target biology, and computational approaches into a unified framework, the review critically discuss representative lead compounds with experimentally validated biological activities and future strategies for clinical translation.

### Literature Search and Study Selection

A focused literature search was conducted to identify studies reporting the synthesis, structural modification, biological evaluation, and SARs of flavones and related flavonoid scaffolds as anticancer agents. Search was performed using platforms such as PubMed, Scopus, Web of Science and Google Scholar, covering publications from approximately 2000 to 2026. The emphasis was on recent studies while retaining earlier landmark SAR investigations. The search keywords used combined terms such as “flavones” or “flavonoids” or “chromones” with “anticancer”, “antiproliferative”, “cytotoxicity”, “structure-activity relationship”, “kinase inhibitors”, “enzyme inhibitors”, “in silico” and “anticancer hybrids”. Studies were considered for inclusion if they (i) investigated flavone or closely related flavonoid/chromone scaffolds, (ii) reported defined structural modifications or comparative analogues enabling SAR interpretation, and (iii) provided experimental anticancer, antiproliferative, target-inhibitory, or mechanistic data with computational studies included to support the experimentally observed SAR trends. Reviews were primarily used to identify relevant studies and provide broader mechanistic context. The original research articles were prioritised for compound-level SAR and biological activity data. Studies lacking sufficient structural or biological information, unrelated flavonoid subclasses, and reports without a meaningful relationship between structural modification and biological outcome were excluded. Priority was given to studies demonstrating clear relationships between functional group substitution, bioisosteric replacement, ring modification, hybridisation, target engagement, and anticancer activity. Representative compounds were therefore selected to illustrate major SAR themes, including hydroxylation and methoxylation patterns, B-heterocyclic rings/C-ring bioisosteric modification, halogen substitution, scaffold extension, molecular hybridisation, and target-directed optimisation. The approach followed enabled integration of medicinal chemistry, biological evaluation, and mechanistic evidence into a focused SAR analysis.

## 2. Cancer Molecular Targets of Flavones: The Role of SARs in Optimising Target Engagement

**Figure 1:**
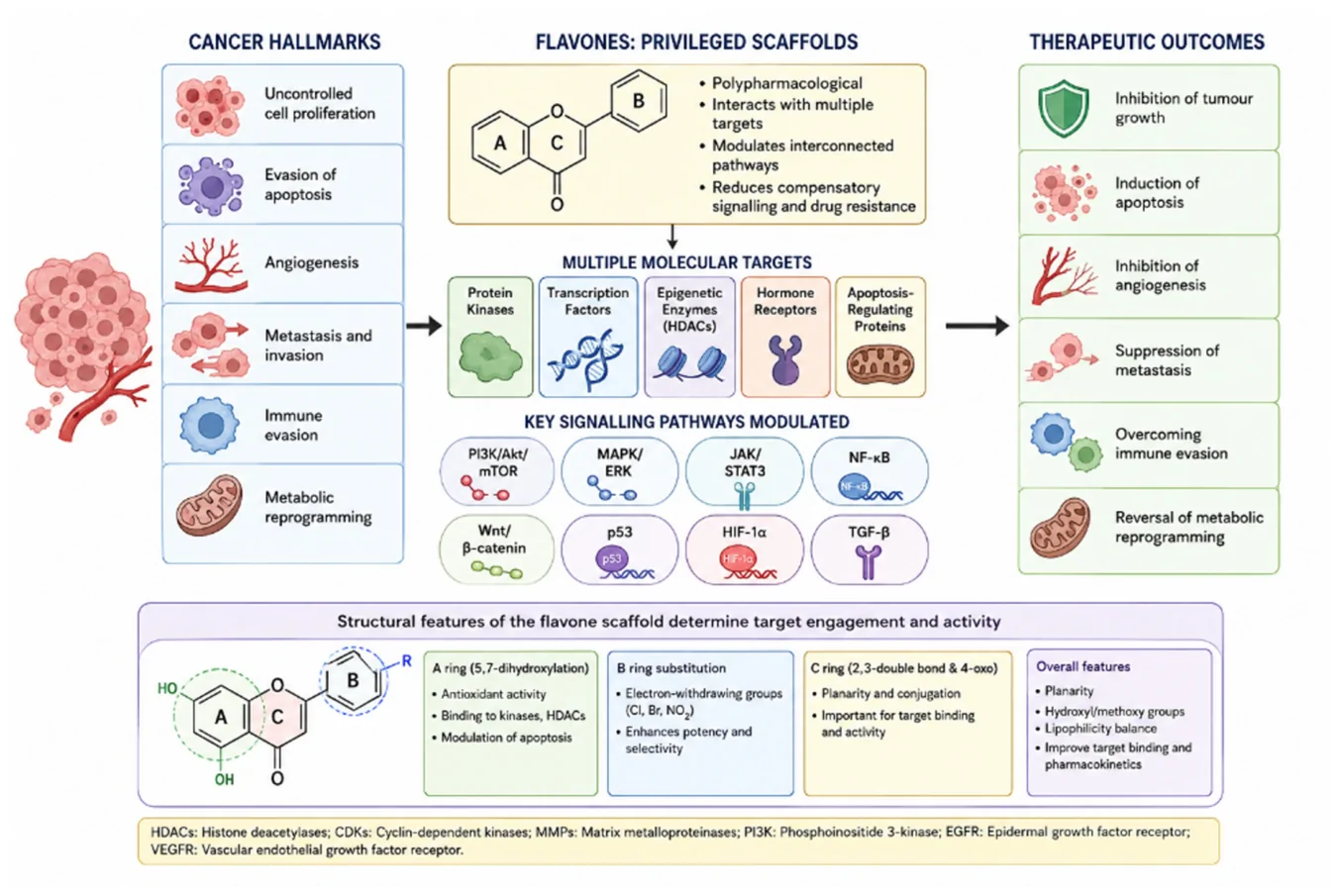
Schematic representation of the multitarget anticancer mechanisms of flavones and the structural determinants governing their interaction with cancer-associated molecular targets. Image created by the authors based on information synthesised from literature.

Cancer is driven by the dysregulation of multiple signalling networks that regulate cell proliferation, apoptosis, angiogenesis, metastasis, immune evasion and metabolic reprogramming as depicted in Figure 1. Flavones have emerged as privileged scaffolds capable of interacting with numerous cancer-associated molecular targets, including protein kinases, transcription factors, epigenetic enzymes, hormone receptors and apoptosis-regulating proteins [3,30]. Their polypharmacological nature enables simultaneous modulation of interconnected signalling pathways such as PI3K/Akt/mTOR, MAPK/ERK, JAK/STAT3, NF-κB, Wnt/β-catenin, p53, HIF-1α and TGF-β, thereby reducing compensatory signalling that frequently contributes to drug resistance [15,23]. Recent studies have also identified flavone derivatives as inhibitors of therapeutically relevant enzymes including histone deacetylases (HDACs), cyclin-dependent kinases (CDKs), topoisomerases (Topos), tubulin, matrix metalloproteinases (MMPs), phosphoinositide 3-kinase (PI3K), epidermal growth factor receptor (EGFR) and vascular endothelial growth factor receptors (VEGFRs), highlighting the versatility of the flavone scaffold for anticancer drug discovery [30,32]. Successful engagement of these molecular targets depends largely on the structural characteristics of the flavone scaffold.

Several SAR studies have demonstrated that subtle modifications of the flavone scaffold, including hydroxylation, methoxylation, halogenation, amination, bioisosteric and hybridisation replacement, profoundly influence target affinity, selectivity and downstream biological responses. These structural changes alter hydrogen-bonding interactions, hydrophobic contacts, electronic distribution, molecular planarity and lipophilicity, ultimately affecting binding within the active or allosteric sites of cancer-associated proteins [30]. Furthermore, SAR-guided optimisation has improved not only potency but also pharmacokinetic properties, metabolic stability, membrane permeability and tumour selectivity, addressing many of the intrinsic limitations associated with naturally occurring flavones [25,32]. Consequently, integrating mechanistic studies with systematic SAR analyses has become a necessary strategy for identifying pharmacophores responsible for target engagement and for developing flavone derivatives with enhanced therapeutic efficacy and reduced off-target toxicity. Building upon these mechanistic insights, the following section systematically examines the SARs of flavone-based anticancer agents from both cell-based and target-based perspectives. Emphasis is placed on how strategic structural modifications around the flavone scaffold influence anticancer potency, target engagement, selectivity and modes of action. Representative lead compounds are highlighted to illustrate the relationship between specific structural features and their biological activities, providing a framework for the rational design and optimisation of next-generation of flavone-derived anticancer therapeutics.

### 2.1. Structural Modification Around Flavone Scaffold for Anticancer Agents (SARs)

Strategic modification of the flavone scaffold through the introduction, removal, or repositioning of functional groups provides an effective approach for optimising interactions with molecular targets involved in cancer initiation, progression and metastasis. Such structural modifications can improve target binding, enhance cellular uptake and metabolic stability, overcome drug resistance, and ultimately increase anticancer potency and selectivity. All structural modulations are keeping the aromatic character of the A- and B-rings essential for their biological activity. As summarised in Table 1 and discussed subsequently, numerous SAR studies have demonstrated that subtle changes in hydroxylation, methoxylation, heteroatom substitution, and ring modifications profoundly influence the biological activity of flavones.

**Figure 2:**
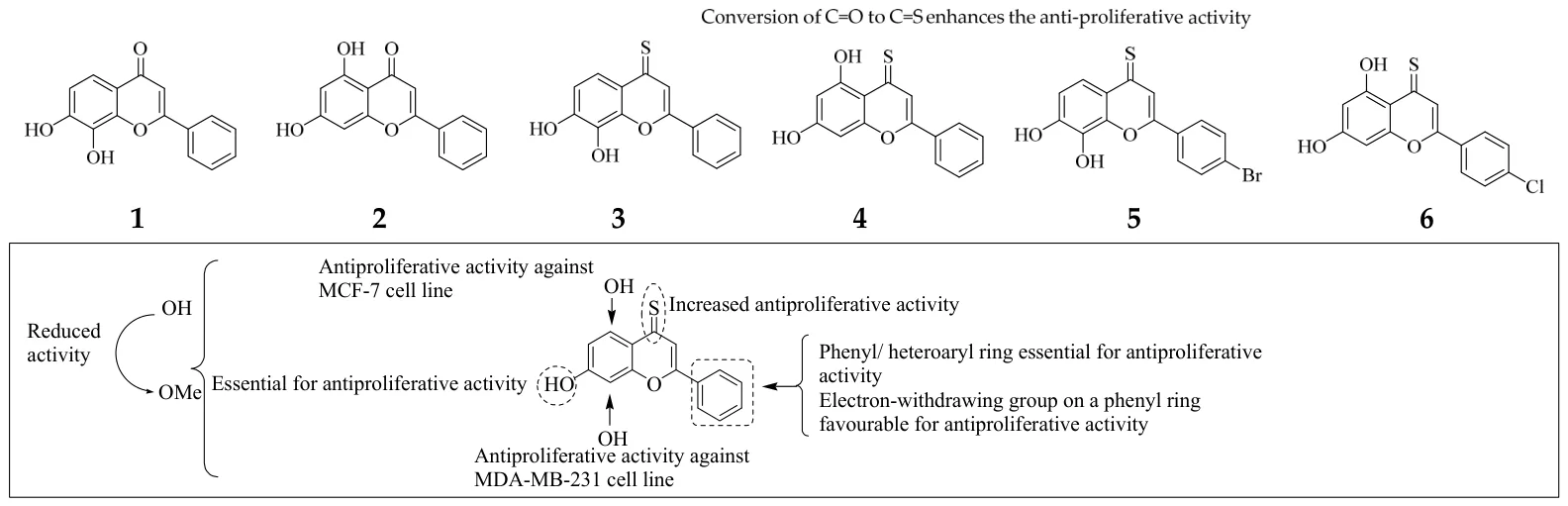
Chemical structures of dihydroxy-substituted flavones (**1** and **2**), 4-thioflavone analogues (**3**–**6**) and SAR analysis.

A library of hydroxy- and methoxy-substituted flavone derivatives, including their 4-thioflavone analogues, was previously synthesised by Ravishankar et al. and, in turn, evaluated against the oestrogen-responsive (MCF-7), doxorubicin-resistant (MCF-7/DX) and triple-negative (MDA-MB-231) breast cancer cell lines using the Methyl Thiazolyl Tetrazolium (MTT) assay [35]. The SAR analysis demonstrated that free phenolic hydroxyl groups are required for activity such as in compounds **1**–**6** (Figure 2). The flavone derivatives **1** and **2** exhibited moderate cytotoxicity against the MCF-7 cell line, with the IC_50_ values of 38.0 µM and 25.6 µM, respectively. These compounds exhibited significantly reduced activity against the MCF-7/DX cell line with the IC_50_ values of 86.7 µM and 176.3 µM, respectively. The thioflavone analogous **4**, **5** and **6**, on the other hand, exhibited significant cytotoxicity against the MCF-7 cell line with the IC_50_ values of 7.9 µM, 4.9 µM and 1.0 µM, respectively. Bioisosteric replacement can substantially modify the physicochemical properties of a molecule, including electronic distribution, bond characteristics, size, lipophilicity, and hydrogen-bonding capacity [36,37,38]. The bioisosteric replacement of the carbonyl oxygen atom of the flavone scaffold with sulphur atom alters electronic, structural and physicochemical properties, primarily owing to the larger size and polarizability of sulphur relative to oxygen [39]. This modification generated the thioflavones **3**–**6** which significantly enhanced antiproliferative potency against both the test cancer cell lines. The 7,8-dihydroxythioflavone **3** exhibited reduced cytotoxicity compared to the isomeric 5,7-dihydroxythioflavone **4** against the MCF-7 (IC_50_ = 14.7 µM and 7.9 µM, respectively) and MCF-7/DX cell line (IC_50_ = 47.2 µM and 34.9 µM, respectively). Incorporation of a lipophilic electron-withdrawing bromine or chlorine atom at the *para* position of the B-ring of **3** or **4** resulted in improved potency for the corresponding derivatives **5** and **6** against the MCF-7 (IC_50_ = 4.9 µM and 1.0 µM, respectively) and the MCF-7/DX cell line (IC_50_ = 6.5 µM and 9.1 µM, respectively). Compounds **5** and **6** with increased cytotoxicity against the MCF-7 and MCF-7/DX cell lines were also evaluated for antigrowth effect against the triple-negative breast cancer cell line, MDA-MB-231. The 2-(4-bromophenyl)-substituted derivative exhibited higher cytotoxicity against this cell line compared to the 2-(4-chlorophenyl)-substituted analogue **6** with the IC_50_ values of 8.9 µM and >10 µM, respectively. The enhanced activity of the 2-(4-halogenophenyl)-substituted derivatives **5** and **6** correlated with increased calculated lipophilicity (LogP = 3.67 and 3.40, respectively) suggesting improved cellular uptake and target interactions. Mechanistic and molecular docking analyses revealed that the growth-inhibitory effects of flavones **5** and **6** are likely driven by an ER-independent mechanism involving enhanced poly(ADP-ribose) polymerase (PARP) cleavage and reduced GSK-3β expression in MCF-7 and MCF-7/DX breast cancer cells. SAR indicated that the position of the hydroxyl groups on the A-ring strongly influence biological activity. Their methylated analogues, on the other hand, generally exhibited reduced antiproliferative properties. Likewise, replacement of the B-ring of the flavone core with a heteroaromatic bioisostere such as thiophene, furan or pyridine ring, for example, significantly reduced activity against the test cancer cell lines, highlighting the importance of hydrophobic and aromatic-aromatic (e.g., π–π) interactions associated with the phenyl ring. The antiplatelet efficacies of the 7,8-dihydroxy and 7,8-dimethoxyflavones and the analogous thioflavones were analysed using human isolated platelets upon activation with cross-linked collagen-related peptide by optical aggregometry [40]. SAR studies revealed that the hydroxyl groups in flavonoids are important for optimum platelet inhibitory activities. The modification of C=O to C=S in the C-ring, and modification of phenyl group (B-ring) into heterocyclic bioisostere such as furan-2-yl, thiofuran-2-yl, thiophen-2-yl or pyridine-3-yl group are tolerated without any significant loss of inhibitory activities.

**Figure 3:**
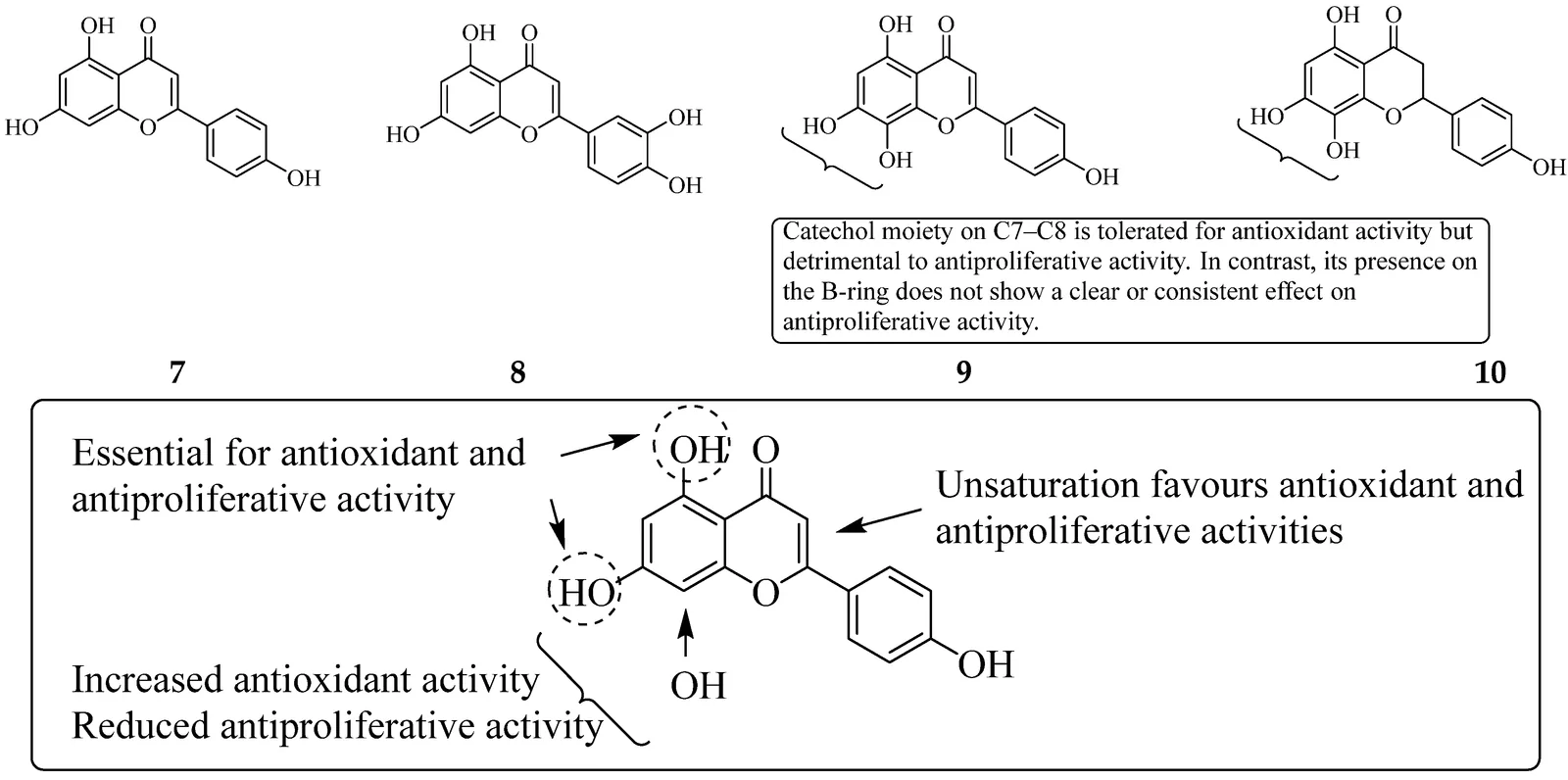
Chemical structures of polyhydroxy-substituted flavones **7**–**10** and SAR analysis.

The significance of hydroxylation patterns was further demonstrated in a study, which compared the naturally occurring flavones and their corresponding flavanone analogues. A series of plant-based flavones substituted with a hydroxyl group at the C-7 or C-7 and C-8 positions, and their flavanone derivatives were investigated for antiproliferative properties and for their antioxidant activity [41]. SAR study revealed that the flavone scaffold was generally more favourable for antiproliferative activity compared to the corresponding flavanone analogues (see selected examples in Figure 3, Table 2). Among the compounds tested, derivatives **7** and **8** exhibited the strongest antiproliferative activity, with the greatest sensitivity observed against the human biphenotypic B myelomonocytic leukaemia (MV-4-11) and human colon cancer (LoVo) cell lines. These flavones displayed IC_50_ values of about 8–9 μM against MV-4-11 cells and 14–15 μM against LoVo cells, highlighting their potential as lead antiproliferative agents. However, the presence of a catechol moiety at the C7–C8 positions of flavone **9** and flavanone **10** was detrimental to the antiproliferative activity. Flavonoids bearing this hydroxylation pattern showed little or no activity across most of the cancer cell lines tested. On the other hand, the SAR studies showed flavones and flavanones containing a C7–C8 catechol-type arrangement or additional hydroxylation generally exhibited enhanced antioxidant properties compared to the C-7 hydroxy-substituted analogues. This observation was attributed to improved hydrogen bond donor effect and stabilisation of the resulting phenoxy radical. Furthermore, the presence of the C(2)-C(3) double bond on the framework of the flavone derivatives contributed to higher antioxidant activity compared to the corresponding flavanones, which lack this unsaturation [41]. These observations illustrate that hydroxyl substitution patterns not only influence direct cytotoxicity but can also determine interactions with biologically relevant enzymes, including the cytochrome P450 isoforms implicated in carcinogenesis [42].

**Figure 4:**
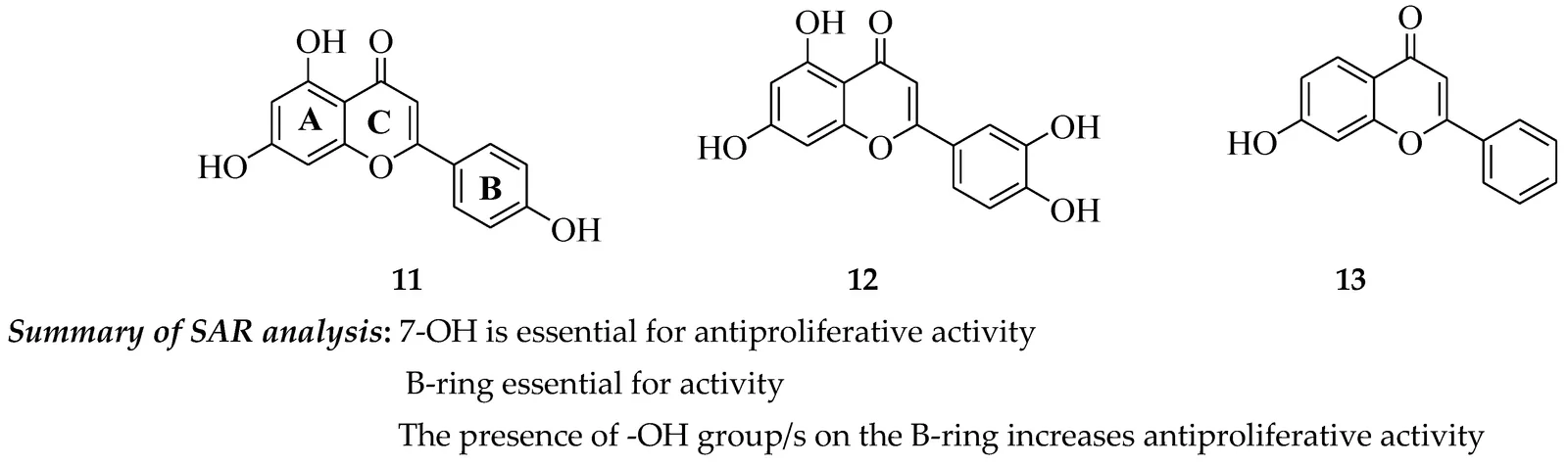
Chemical structures of the most active flavone-based AKR1B10 inhibitors **11**–**13** and summary of SAR analysis.

Zemanova et al. studied the naturally occurring phenolic compounds and flavones as a novel class of aldo-keto reductase family 1 member B10 (AKR1B10) inhibitors, a nicotinamide adenine dinucleotide phosphate (NADPH)-dependent reductase that is highly overexpressed in several cancers, including hepatocellular, lung, pancreatic, and breast cancers [43]. The study highlighted apigenin **11**, luteolin **12** and 7-hydroxyflavone **13** shown in Figure 4 as the most active against AKR1B10 amongst the tested compounds compared to the positive control, flufenamic acid (IC_50_ = 96.0 µM) with the IC_50_ values of 6.6 µM, 9.2 µM, and 8.3 µM, respectively. However, these compounds exhibited moderate selectivity against AKR1B1 cells with the IC_50_ values of 11.9 µM, 3.2 µM and 12.3 µM respectively, indicating a preference for the cancer-associated isoform. Compounds **11**, **12** and **13** inhibited daunorubicin metabolism with the IC_50_ values of 37.1 µM, 107.9 µM and 160.0 µM, respectively. SAR analysis revealed that hydroxyl substitution at the C-7 position of the flavone scaffold and the presence of hydroxyl group/s on the B-ring were crucial for activity. Molecular docking demonstrated that the presence of the hydroxyl groups promotes hydrogen-bonding and hydrophobic interactions within the AKR1B10 active site. Moreover, the lead flavones inhibited AKR1B10-mediated daunorubicin metabolism in intact cells without significant cytotoxicity, supporting their potential as templates for the development of selective anticancer agents targeting AKR1B10.

**Figure 5:**
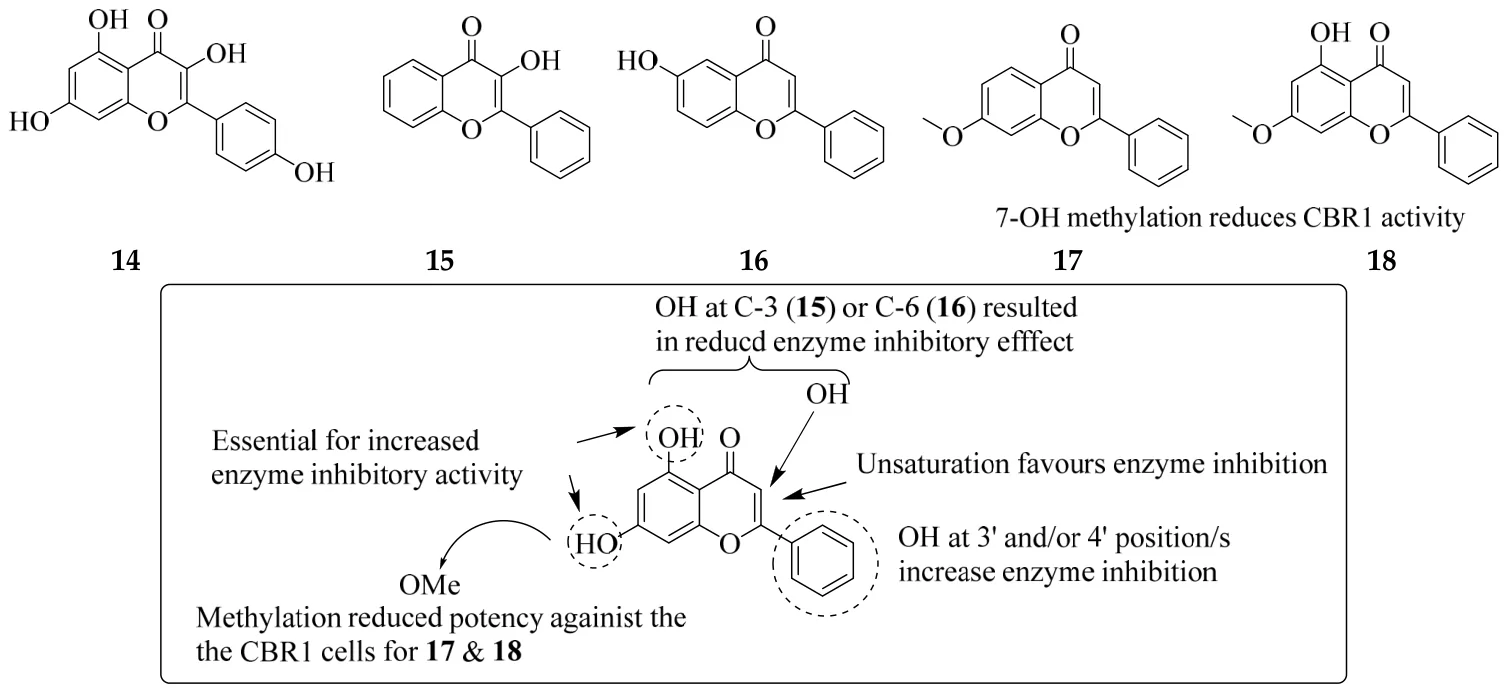
Chemical structures of the hydroxylated flavone derivatives **14**–**18** and a summary of SAR analysis.

The significance of hydroxylation patterns was also demonstrated against another clinically relevant enzyme, carbonyl reductase 1 (CBR1), which catalyses the conversion of anthracycline anticancer drugs such as doxorubicin into less active but more cardiotoxic metabolites [44]. Since inhibition of CBR1 may enhance anthracycline efficacy while reducing cardiotoxicity, a study was conducted to evaluate flavonoids as CBR1 inhibitors [45]. Among the flavonoid subclasses examined, flavones exhibited the strongest inhibitory potency against CBR1, with apigenin **11** (IC_50_ = 0.09 µM) and luteolin **12** (IC_50_ = 0.13 µM) identified as the lead compounds. The activity of kaempferol **14**, a flavonol analogue shown in Figure 5 was comparable to that of luteolin with an IC_50_ value of 0.13 µM. The monohydroxylated flavones **15** and **16** bearing a hydroxyl group at the C-3 or C-6 position of the 2-phenylchromen-4-one scaffold respectively, resulted in markedly reduced activity with the IC_50_ values greater than 10 μM. Methylation of the C-7 hydroxyl group in compound **17** and tectochrysin **18** also substantially reduced potency against the CBR1 cells with % inhibition values of 37% and 9.6% at 10 µM, respectively. SAR analysis revealed that the unsaturation at the C2-C3 position, together with hydroxyl substituents at C-5 and C-7 and additional hydroxyl groups at the 3′- and 4′-positions of the B-ring were essential for potent enzyme inhibition. Kinetic studies further demonstrated that these flavone analogues directly interact with the catalytic site of CBR1 through complementary hydrogen-bonding and hydrophobic interactions, emphasising the importance of maintaining the planar flavone framework.

**Figure 6:**
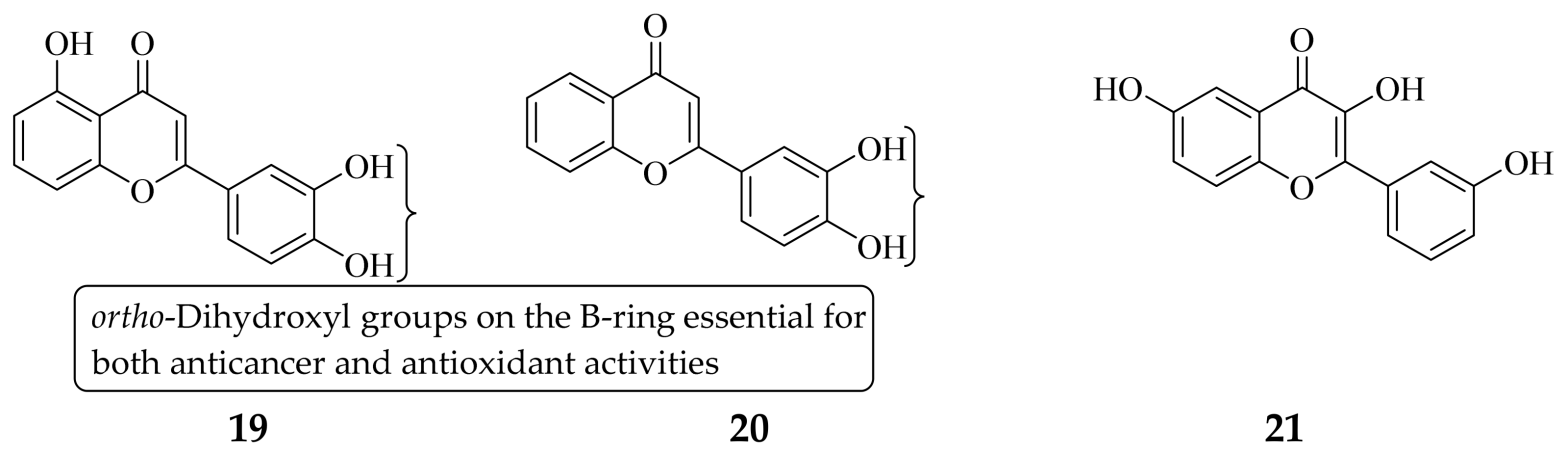
Chemical structures of representative A- and B-ring hydroxylated flavones **19**–**21**.

Grigalius and Petrikaite previously examined the relationship between hydroxyl substitution and biological (antioxidant and antiproliferative) activity of seventeen trihydroxyflavone derivatives [46]. Majority of the tested trihydroxyflavones exhibited antiproliferative activity, with breast cancer cells (MCF-7) being the most sensitive and glioblastoma cells (U87) the least responsive. The lead compound 3′,4′,5-trihydroxyflavone **19** shown in Figure 6 displayed the strongest cytotoxicity against both the A549 (IC_50_ = 6.8 µM) and MCF-7 (IC_50_ = 11.2 µM) cells compared to apigenin (IC_50_ = 77.5 µM and 68.2 µM, respectively) and baicalein (IC_50_ = 71.5 µM and 26.1 µM, respectively) used as the reference standards for the assay. Although less active against the A549 and MCF-7 cell lines, derivative **20** exhibited significant activity against the U87 cell compared to **19** with the IC_50_ values of 15.5 µM and 30.8 µM, respectively. The flavonol analogue **21**, on the other hand, exhibited significantly reduced activity against the A549 cells (IC_50_ = 36.3 µM), but was the most active against the MCF-7 and U87 cell lines with the IC_50_ values of 2.6 µM and 14.2 µM, respectively. SAR investigation showed that the *ortho*-dihydroxyl arrangement (3′,4′-OH) on the B-ring is the key pharmacophore for both anticancer and antioxidant activities. Compounds bearing this motif, particularly compounds **19** and **20** exhibited the strongest antioxidant effects and enhanced antiproliferative activity. Despite exhibiting strong antiproliferative activity against the MCF-7 and U87 cell lines, the flavonol analogue **21** which lacks the 3′,4′-OH motif showed poor antioxidant activity. The calculated Pearson coefficient, on the other hand, showed poor correlation between the anticancer and antioxidant activities.

**Figure 7:**
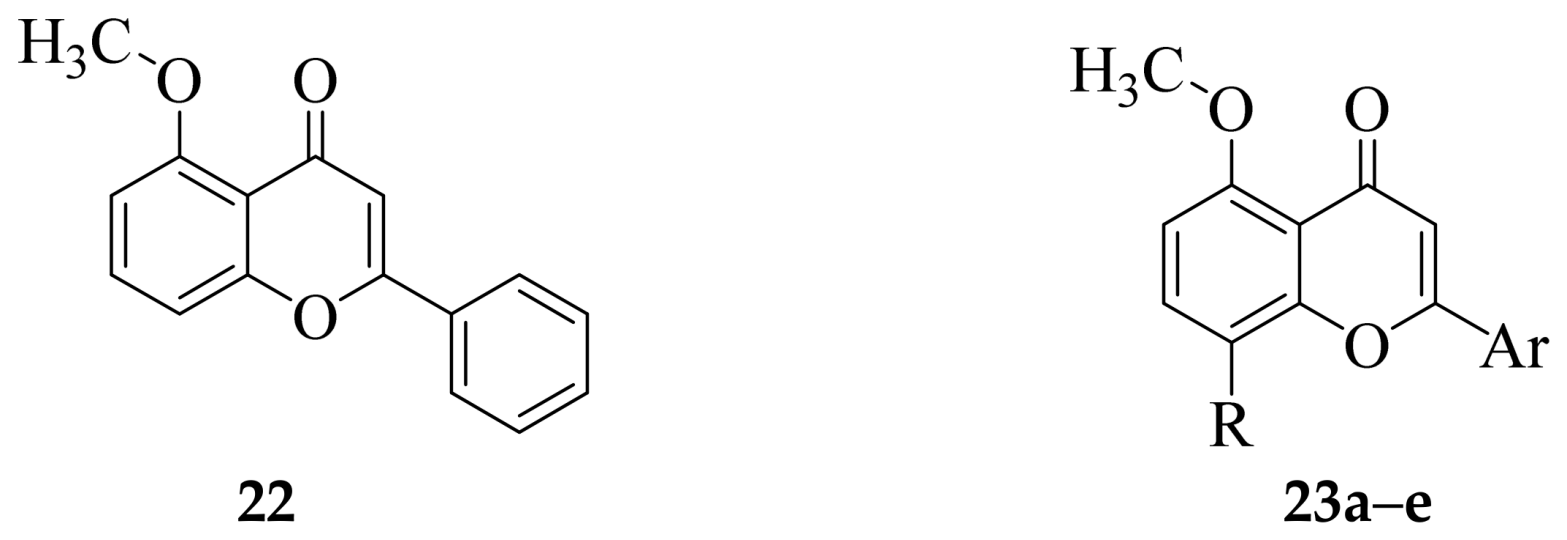
Chemical structures of 5-methoxyflavone **22** and the 8-carbo-substituted flavones **23**.

Early SAR studies established the hydroxyl group substitution pattern, co-planarity and the bioisosteric modifications of the 2-phenylchromen-4-one scaffold to be important for the biological activity of flavones. More recent studies have employed rational scaffold optimisation to further improve anticancer efficacy. The presence of methoxy group/s on the flavone scaffold, for example, was found to promote cytotoxic activity in various cancer cell lines by targeting protein markers to enhance membrane-penetrating properties leading to facilitated absorption and highly increased oral bioavailability [19,47]. A SAR study on the biological activity of the A-ring monomethoxy-substituted flavones against leukemic HL60 cells previously showed that the presence and position of the methoxy group has a significant effect on antiproliferative activity and the trend was, 5-methoxy- (48 μM) > 7-methoxy- (68 μM) > 6-methoxyflavone (400 μM) [48]. 5-Methoxyflavone **22** shown in Figure 7 has also been found to exhibit anti-inflammatory properties, vasodilatory and antiproliferative activities [49]. The 8-aryl-, 8-styryl- and 8-arylethynyl-substituted 5-methoxyflavones **23** were synthesised and evaluated for anti-breast and anti-lung cancer activity targeting protein kinases (VEGFR-2 and EGFR) [50]. Compounds **23a**, **23b**, **23c**, **23d** and **23e** exhibited strong activity against the MCF-7 cell line (IC_50_ = 13.68 ± 0.72, 16.91 ± 0.40, 13.63 ± 0.36, 14.66 ± 0.47 and 12.26 ± 0.45 μM, respectively) and lung (A549) cancer cell line (IC_50_ = 15.38 ± 0.33, 10.00 ± 0.28, 12.38 ± 0.30, 12.84 ± 0.33 and 8.47 ± 0.30 μM, respectively) among the test compounds compared to quercetin (IC_50_ = 40.61 ± 1.07 and 58.17 ± 0.50 μM, respectively). The presence of a small phenyl ring or halogenophenyl group linked to the C-8 position of the A-ring directly or through a π-conjugated bridge seemed to be desirable for the cytotoxicity of the 8-carbo-substituted 5-methoxyflavones. Compounds **23b**, **23c** and **23e** exhibited dual inhibitory effect against VEGFR-2 and EGFR tyrosine kinase phosphorylation (Table 3). Molecular docking revealed that strong alignment with the enzyme backbone is achieved mostly by hydrophobic (π-π, and π-H) contacts and by hydrogen-bonding interaction with the residues in the active sites of VEGFR-2 and EGFR. The orientation of the methoxy group of these compounds facilitated hydrogen bonding with Cys919 in the hinge region of VEGFR-2. The geometry about the π-conjugated (C–C) bridge of **23b** enabled the 4-fluorophenyl ring of this compound to engage in hydrogen-bonding interaction with Val848 in the front pocket. er contributing to the inhibitory effect of this compound against VEGFR-2. This residue is also involved n-π interaction with the chromone framework further contributing to the inhibitory effect of this compound against VEGFR-2. The most active derivative **23c** against EGFR forms a strong hydrogen-bonding interaction with the key residue Met769, along with dual n-π interactions with Val702, Arg817 and Leu694 as well as single n-π interaction with Thr830 and Cys773. The test compounds possess favourable drug-likeness properties, supporting their potential as promising therapeutic candidates.

**Figure 8:**
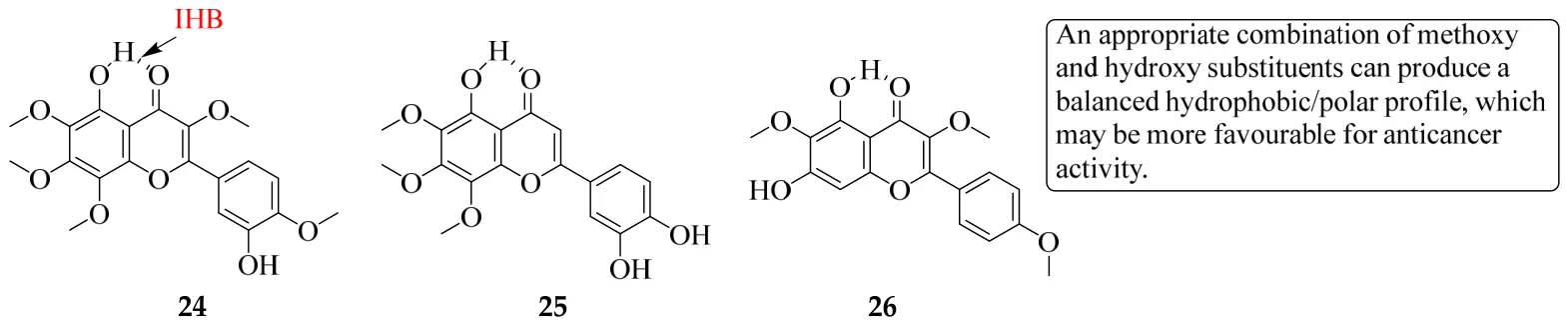
Chemical structures of the polymethoxyflavones **24**, **25** and **26**.

A review by Aidiel et al. on the mechanistic insights into the anticancer potential of methoxyflavones analogues, for example, explored the synergistic role of hydroxy and methoxy moieties through hydrogen-bonding capacity in maximising cytotoxicity against various cancer cell lines [19]. Compounds such as 3,6,7,8,4′-pentamethoxyflavone **24** and sideritoflavone **25** shown in Figure 8 were identified as the most potent among the test series, showing MCF-7 breast cancer cells IC_50_ values of 3.71 and 4.9 μM, respectively. Compound **26**, on the other hand, was identified as the most potent against the A2058 melanoma cell line with an IC_50_ value of 3.92 μM. SAR studies revealed that the methoxy groups occupied at least two out of three positions from the C6, 7 and 8 positions with the C7-OCH_3_ resulting in strong cytotoxic activity of the methoxyflavone analogues. The C7-OH could exert a similar effect with the addition of at least one C6-OCH_3_ group. The proximity of the C5 hydroxyl group to the carbonyl group facilitates intramolecular hydrogen-bonding interaction (IHB) to form a six-membered ring-like motif (see Figure 8) that mimics the topology of a heteroaromatic or heterocyclic ring present in the classical kinase inhibitors [51]. Increased co-planarity due to the formation of this pseudo-aromatic ring-like motif is fundamental to stronger anticancer activity of the flavone derivatives. The resultant locked conformation in the case of kinase inhibitors results in favourable alignment of drug molecules with the protein pocket to interact with crucial amino acid residues within the catalytic ATP-binding domain [52]. The presence of additional hydroxy moieties alongside the methoxy group on either the C3′ or C4′ position could amplify the cytotoxicity activity by the resonance effect of free radicals which contribute to greater hydrogen bond donor capacities. A balance between the number and position of the methoxy and hydroxy groups on ring A and B of flavones was found to be crucial to maximise interaction with the target protein marker. The collaborative efficacy of both groups maximises the resonance effect and electron delocalisation of free radical flavones and initiates the formation of intramolecular hydrogen bonds. Concurrently, both interaction mechanisms stabilised and preserved the lipophilicity while simultaneously offering an optimal polar surface for stronger interaction with the cancer cell death marker.

**Figure 9:**
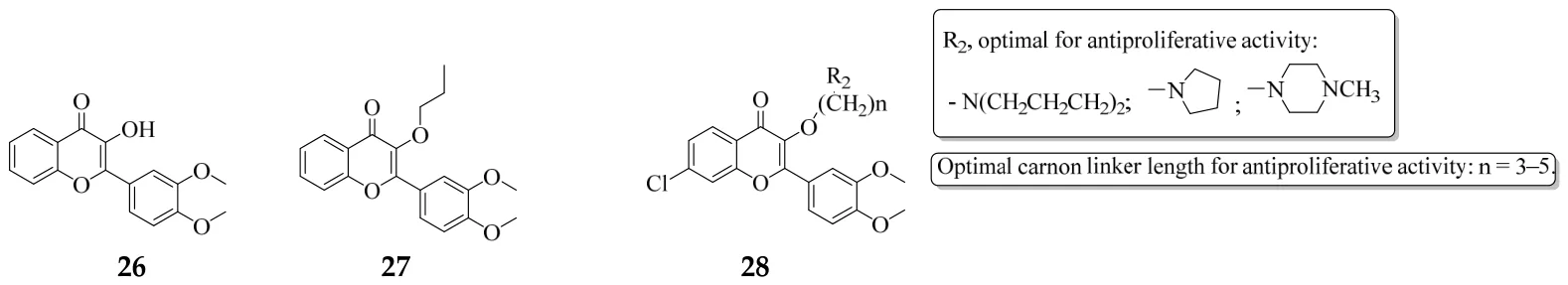
Chemical structures of the 3-*O*-substituted flavones.

A series of the 3-alkoxy-substituted 3′,4′-dimethoxyflavonols was synthesised and evaluated for antiproliferative activity against androgen-independent PC-3 and androgen-sensitive LNCaP prostate cancer cell lines using the water-soluble tetrazolium salt 1 (WST-1) assay [53]. All the synthesised derivatives exhibited greater antigrowth effect against this cancer cell line compared to the parent compound **26** (Figure 9) which served as a baseline for this study. Compound **26** showed weak cytotoxicity against the PC-3 and LNCaP cell lines with the IC_50_ values of 409.0 μM and 202.5 μM, respectively. Simple 3-alkoxy-substituted derivatives, on the other hand, exhibited substantially improved antigrowth effect against the PC-3 and LNCaP cells. Compound **27** (R_2_ = -N(CH_2_CH_2_CH_2_)_2_) was identified as a lead amongst the set with the IC_50_ values of 10.1 μM and 6.4 μM against the PC-3 and LNCaP cell line, respectively. The highest enhancement against the PC-3 and the LNCaP cells was achieved with the 3-alkoxyaminodialkyl derivatives with the IC_50_ values in the range 1.4–10.9 µM and 2.4–14.9 µM, respectively. Compound **28** with a dibutylamino group linked to oxygen atom through a three-carbon spacer emerged as the lead candidate with the IC_50_ values of 1.4 μM (PC-3) and 2.4 μM (LNCaP), representing approximately 292-fold and 84-fold improvements compared to compound **26**, respectively. SAR studies revealed that modification of the C-3 hydroxyl group is a key determinant of biological activity. Conversion of the 3-OH to simple alkyl ethers significantly increased antiproliferative potency. Incorporation of basic aminoalkyl side chains, on the other hand, significantly improved activity against the test cell lines. A dibutylamino substituent, for example, was consistently superior to morpholine-containing analogues, suggesting that increased lipophilicity and the presence of a protonatable tertiary amine favour cellular uptake and target interactions. The shorter linkers, e.g., a three-carbon chain, between the flavonol oxygen and dibutylamino group were optimal for activity. Extending the linker to four or five carbons resulted in a gradual decline in activity, indicating that linker length is critical for maintaining favourable molecular geometry. Neither compound **26** nor analogue **28** inhibited proliferations of normal PWR-1E prostate epithelial cells at concentrations up to 50 μM, suggesting promising selectivity and safety profiles at least in vitro. Mechanistic cell-based studies in vitro revealed that compound **28** induced significant G_2_/M cell-cycle arrest and triggered dose-dependent apoptosis in PC-3 cells. These findings demonstrate that strategic modification of the flavonol C-3 position can substantially enhance antiproliferative potency while maintaining favourable selectivity toward normal cells.

**Figure 10:**
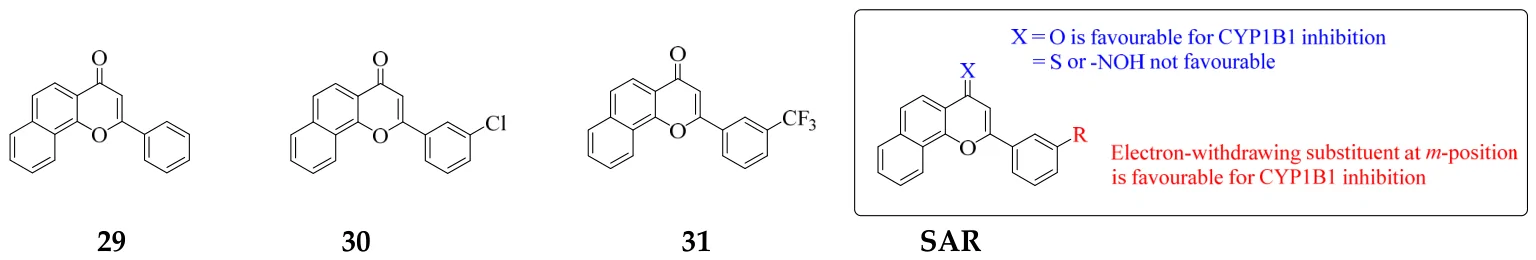
Chemical structures of α-naphthoflavone **29**–**31** and summary of SAR analysis for CYP1B1 inhibition.

Subsequent studies have explored flavone derivatives as selective modulators of molecular targets that are overexpressed in cancer, thereby broadening the therapeutic applications of this scaffold. Dong et al. previously used α-naphthoflavone **29** shown in Figure 10 as the lead scaffold for optimisation owing to its potent cytochrome P450 1B1 (CYP1B1) inhibitory activity and the available crystal structure of this flavone derivatives in complex this enzyme [54]. CYP1B1 is an extrahepatic enzyme that is overexpressed in numerous cancers and is involved in the metabolic activation of procarcinogens and the development of chemoresistance [55]. This enzyme is overexpressed in tumours compared to the normal tissues, and thus its selective inhibition represents an attractive anticancer strategy. A series of α-naphthoflavone analogues was synthesised and evaluated for potential to improve CYP1B1 inhibitory potency and selectivity against the CYP1A1 and CYP1A2 isoforms. Derivatives **30** and **31** were identified as the most potent CYP1B1 inhibitors amongst the tested compounds with the IC_50_ values 0.49 and 0.52 nM, respectively (Figure 10). Both compounds were approximately 10-fold more potent than α-naphthoflavone **29**. These compounds exhibited variable inhibitory activity against CYP1A1 with the IC_50_ values of 80.3 nm, 5.2 nm and 16.7 nm, respectively. Significantly reduced activity of **29**, **30** and **31** was observed against CYP1A2 with the IC_50_ values of 18.0 µM, 73.6 µM and 94.2 µM, respectively. The two lead compounds **30** and **31** were more selective towards CYP1B1 enzyme compared to CYP1A1 and CYP1A2 isoforms with selectivity index values >10 and >150, respectively. Replacement of carbonyl oxygen with a sulphur atom or oxime moiety resulted in reduced activity against CYP1B1. The presence of electron-withdrawing substituent on the *meta* position of the 2-phenyl ring (e.g., Cl, -CF_3_) was found to be optimal for activity. Molecular docking into the crystal structures of CYP1B1 or CYP1A2 in complex with **29** (PDB ID: 3PM0 and 2HI4, respectively) showed that chlorine atom in compound **30** occupies the hydrophobic region of CYP1B1. This hydrophobic region is absent in the isoforms CYP1A1 and CYP1A2, probably accounting for the observed enhanced selectivity against CYP1B1 in vitro.

**Figure 11:**
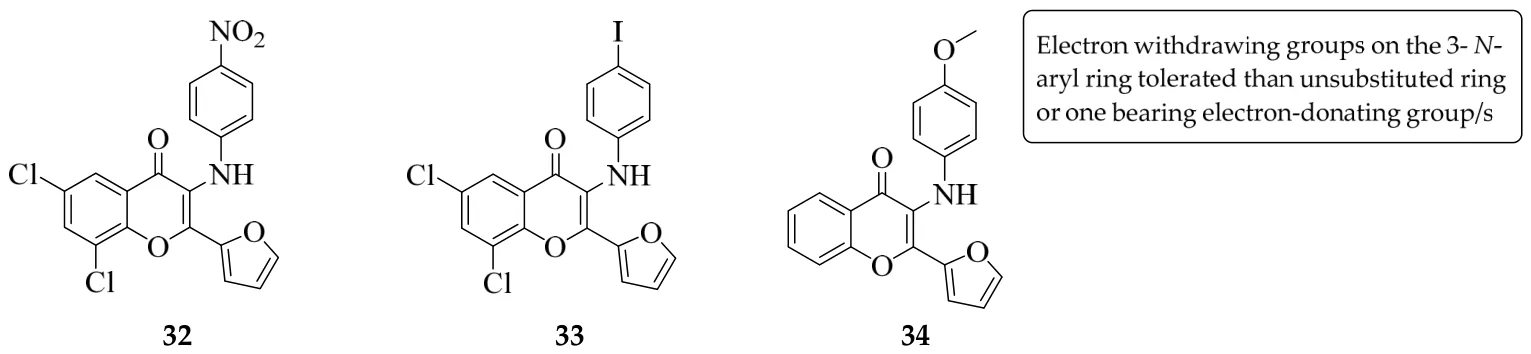
Chemical structures of representative flavone-based MARK4 inhibitors **32**–**34**.

Selective enzyme inhibition represents an attractive strategy for suppressing tumour progression and overcoming chemoresistance. Flavone-based analogues have also been evaluated for inhibitory effects against protein kinases that regulate cancer cell proliferation and metastasis, thus expanding the scope beyond metabolic enzymes to intracellular signalling pathways. Parveen et al. previously synthesised a series of the 3-*N*-aryl-substituted 2-heteroarylchromones and evaluated them as microtubule affinity-regulating kinase 4 (MARK4) inhibitors [56]. MARK4 is a serine/threonine kinase implicated in cancer progression, metastasis, microtubule dynamics and neurodegenerative disorders [57]. The study integrated enzymatic inhibition assays, molecular docking, and cellular antiproliferative studies to identify novel MARK4 targeting agents. Several compounds exhibited potent kinase inhibition in the low micromolar range. Among the series, compounds **32**, **33** and **34,** shown in Figure 11 containing a *para*-substituent on the *N*-aryl ring, showed superior activity against MARK4 with the IC_50_ values of 2.12 μM, 1.98 μM and 5.56 μM, respectively. These compounds also showed significant antiproliferative activity against the MCF-7 cell line (IC_50_ = 3.22 μM, 4.32 μM and 16.22 μM, respectively) and the human liver carcinoma (HepG2) cell line (IC_50_ = 6.45 μM, 5.22 μM and 19.12 μM, respectively), supporting the role of MARK4 inhibition in suppressing tumour cell growth. The SAR investigation revealed the chromone scaffold as essential for MARK4 inhibitory activity. The presence of electron-withdrawing groups on the *N*-aryl ring, on the other hand, enhanced potency compared to the unsubstituted or electron-donating analogues. Docking studies demonstrated that the lead chromones **32**, **33** and **34** fit well within the ATP-binding site of MARK4. Key interactions included hydrogen bonding with residues lining the catalytic region, aromatic-aromatic (π–π stacking) interactions between the chromone ring system and aromatic amino acids as well as extensive hydrophobic contacts that stabilised ligand binding. These computational (in silico) results correlated well with the observed in vitro activities and provided a rationale for the SAR trends. Collectively, these findings demonstrate the versatility of the chromone/flavone scaffold for targeting intracellular enzymes involved in tumour progression.

**Figure 12:**
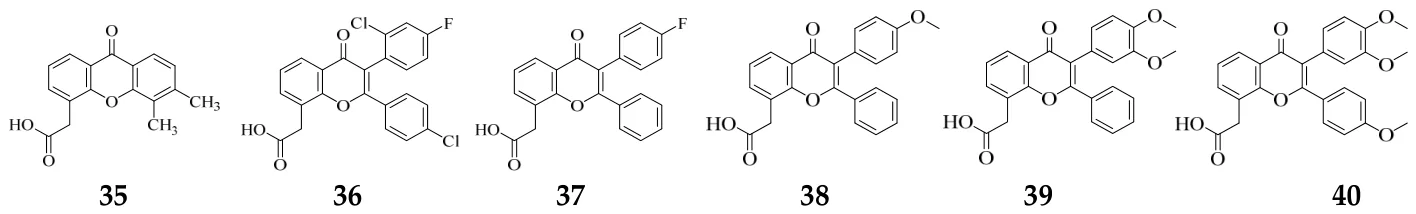
Chemical structures of vadimezan **35** and representative flavone analogues **36**–**40.**

Flavone-based drug design has also been directed toward agents that exert antitumour activity through modulation of the tumour microenvironment rather than direct inhibition of intracellular targets. Yan et al. designed and synthesised a series of novel 3-arylflavone-8-acetic acid derivatives as analogues of the anticancer agent, vadimezan **35** shown in Figure 12 [58]. Vadimezan is known to exert its anticancer effects primarily by stimulating cytokine production and disrupting tumour vasculature and this compound has progressed to phase III clinical evaluation as a vascular-disrupting agent with a favourable safety profile in humans [59]. The study was aimed to elucidate the influence of substituents on both the 3-aryl ring and the flavone scaffold on anticancer activity, thereby establishing SARs to guide the development of more potent antitumour agents. The synthesised compounds were evaluated for direct and indirect antiproliferative activity against the human colon adenocarcinoma (HT-29) and lung A549 cancer cell lines, as well as for toxicity toward human peripheral blood mononuclear cells (HPBMCs). SAR analysis revealed that compounds bearing electron-withdrawing substituents (e.g., halogen atoms) generally displayed moderate direct cytotoxic effects with compound **36** (Figure 12) showing more activity against the A549 and HT-29 cells (IC_50_ = 239 and 217 µM, respectively) compared to **35** (IC_50_ = 291 and 268 µM, respectively) as shown in Table 4. Compounds **36** and **37**, on the other hand, exhibited indirect cytotoxic activities comparable to those of **32**, while maintaining low toxicity toward HPBMCs. Notably, analogues containing methoxyphenyl substituent groups at the C-2 or C-3 positions of the flavone nucleus demonstrated stronger indirect cytotoxicity against A549 cells than **35** with compounds **38**–**40** (Figure 12) showing approximately 2-fold improved activity. These compounds further showed reduced toxicity toward HPBMCs. Furthermore, compounds **38**–**40** could stimulate tumour necrosis factor-α (TNF-α) production in HPBMCs, suggesting that their anticancer activity may be mediated, at least in part, through immunomodulatory mechanisms. These results illustrate that appropriate substitution of the flavone scaffold can enhance indirect antitumour activity by stimulating cytokine production while reducing toxicity toward normal cells.

**Figure 13:**
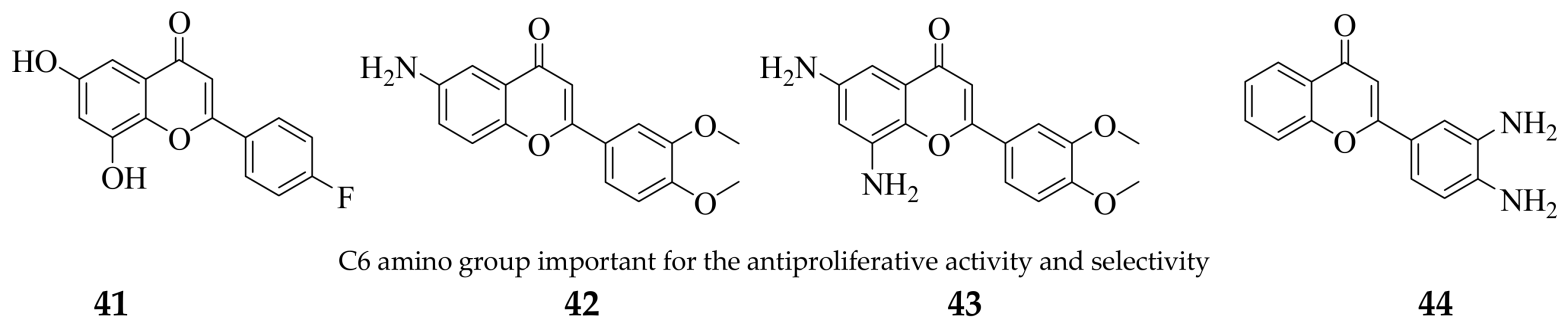
Chemical structures of flavone **41** and aminoflavone derivatives **42**–**44**.

Recent studies have further explored flavone derivatives as modulators of innate immune responses, particularly through enhancement of natural killer (NK) cell-mediated tumour cytotoxicity. Flavones and their amino-substituted derivatives have demonstrated significant potential as immunomodulatory anticancer agents by enhancing NK cell-mediated cytotoxicity against non-small cell lung cancer (NSCLC), offering an alternative to conventional direct cytotoxic therapies. Hsu et al. evaluated synthetic flavones and aminoflavones against A549 (EGFR wild-type) and H1975 (EGFR T790M/L858R) NSCLC cell lines using NK-92MI cells as effector cells [60]. Flavone **41** together with aminoflavones **42**–**44** (Figure 13, Table 5) significantly enhanced NK cell-mediated lysis of A549 cells while exhibiting minimal toxicity toward normal MRC5 lung fibroblasts, indicating favourable selectivity. Aminoflavone **42** was the lead compound, displaying moderate antiproliferative activity against the A549 (IC_50_ = 23.14 μM; selectivity index = 3.03) and H1975 cells (IC_50_ = 38.65 μM). Flavone **41**, on the other hand, exhibited comparable activity against the A549 cells (IC_50_ = 24.20 μM), while compound **44** showed lower potency (IC_50_ = 34.38 μM). Mechanistically, aminoflavone **42** enhanced NK cell activation by increasing the production of interferon-γ (IFN-γ), perforin, and granzyme B, while simultaneously suppressing STAT3 phosphorylation in both NK-92MI and A549 cells, thereby promoting immune-mediated tumour cell killing. A combination of aminoflavone **42** and NK-92MI cells produced synergistic tumour growth inhibition in an A549 xenograft model without detectable hepatic or renal toxicity. SAR studies identified the C6 amino substituent as the critical pharmacophore responsible for enhanced NK cell activation, with amino substitution markedly improving immunomodulatory efficacy over the parent flavone scaffold while maintaining low toxicity toward normal cells. Collectively, these studies demonstrate that rational structural modification of the flavone scaffold enables modulation of diverse anticancer mechanisms, including direct cytotoxicity, kinase inhibition, enzyme selectivity, tumour vascular disruption, and immune activation.

**Figure 14:**
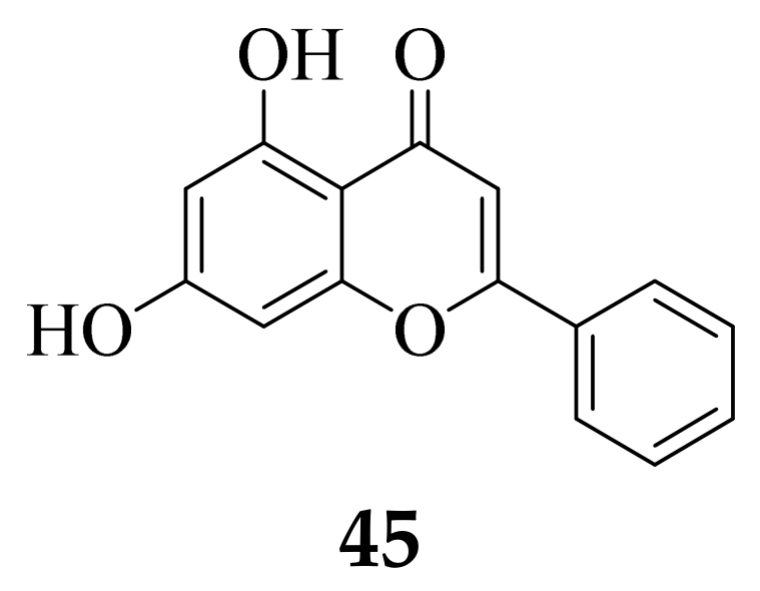
Chemical structure of the naturally occurring chrysin.

A recent systematic review demonstrated that numerous dietary phytochemicals, including polyphenols, flavonoids, stilbenes, isothiocyanates, alkaloids, and vitamins, regulate DNA methylation through direct or indirect modulation of DNA methyltransferases (DNMTs) activities [61]. DNMTs, particularly C5-DNA methyltransferases catalyse the transfer of a methyl group to the C5 position of cytosine residues, thereby regulating gene expression, chromatin organisation, and genome stability. Dysregulated DNA methylation is a hallmark of numerous human diseases, particularly cancer, making DNMTs attractive therapeutic targets for epigenetic intervention [62,63,64,65,66]. Although the nucleoside analogues azacitidine and decitabine have demonstrated clinical efficacy as DNMT inhibitors, their poor chemical stability, limited selectivity, and dose-limiting toxicities have stimulated considerable interest in identifying safer and more selective non-nucleoside inhibitors. Natural products have emerged as an important source of DNMT modulators. Among the most extensively investigated compounds are epigallocatechin-3-gallate (EGCG), curcumin, genistein, resveratrol, sulforaphane, folate, berberine, and sodium butyrate, many of which inhibit DNMT1, DNMT3A, and DNMT3B in a dose- and time-dependent manner. Notably, nearly 90% of the preclinical studies included in the review [61] reported significant DNMT inhibitory activity, highlighting dietary bioactive compounds as promising epigenetic modulators with potential applications in healthy ageing, cancer prevention, and other chronic diseases. There has been increasing attention directed towards exploring flavones as epigenetic modulators, particularly through inhibition of DNMTs, thereby expanding the therapeutic potential of this privileged scaffold beyond conventional signalling pathways. Among them, the naturally occurring flavones apigenin **11** and luteolin **12,** represented in Figure 4, as well as chrysin **45**, shown in Figure 14, have received particular attention as dual epigenetic modulators capable of simultaneously inhibiting DNMTs and histone methyltransferases (HMTs), particularly enhancer of zeste homolog 2 (EZH2), the catalytic enzyme responsible for H3K27 trimethylation [67]. Unlike conventional nucleoside DNMT inhibitors, these flavones act through a non-nucleoside mechanism while exhibiting relatively low toxicity and the ability to simultaneously modulate DNA and histone methylation. Luteolin **12** emerged as the lead compound, producing the strongest inhibition of DNMT and HMT activities, and the largest reductions in EZH2 expression and H3K27 trimethylation. Apigenin **11**, on the other hand, displayed intermediate potency, whereas chrysin **45** was the least active against these biochemical targets. SAR analysis revealed that hydroxyl substitution on the B-ring is the principal determinant of epigenetic activity. Compound **12**, possessing a 3′,4′-dihydroxy substitution pattern, exhibited the highest DNA-binding affinity and strongest inhibition of both DNMTs and EZH2. Apigenin **11**, containing only a single 4′-hydroxyl group showed moderate activity, while chrysin **45** lacking B-ring hydroxyl substituents displayed the weakest DNA intercalation and enzyme inhibition. Molecular docking studies demonstrated that these hydroxyl groups establish hydrogen-bonding interactions within the catalytic pockets of DNMT1 and EZH2, thereby enhancing binding stability. Furthermore, all three flavones preferentially intercalated into GC-rich DNA sequences, inhibited methylation of the GSTP1 promoter and promoted DNA demethylation potentially restoring tumour suppressor gene expression.

**Figure 15:**
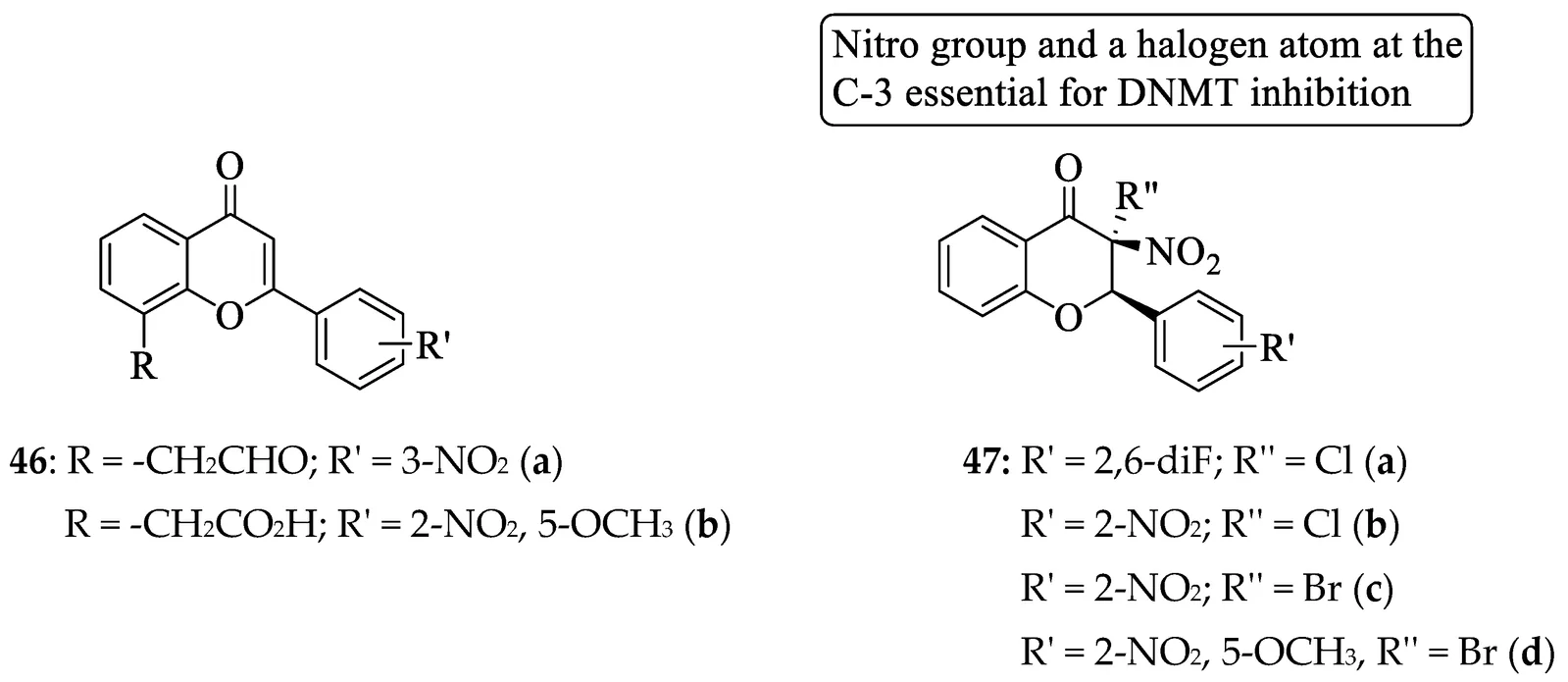
Chemical structures of the flavone derivatives **46** and 3-halo-3-nitroflavanone derivatives **47**.

The promising anticancer activity of naturally occurring flavones as potential DNMT inhibitors has inspired the development of synthetic flavonoid-based analogues with improved potency. High-throughput screening efforts previously identified several structurally diverse scaffolds with inhibitory activity against C5-DNMTs [68]. The study identified 3-nitroflavones as promising DNMT inhibitors with compounds **46a** and **46b** shown in Figure 15 as early leads showing DNMT3A/3L IC_50_ values of 9.50 and 1.26 µM, respectively. Interestingly, the hydrohalogenation of the C2=C3 moiety to yield the 3-halo-3-nitroflavanones generally resulted in an increased DNMT3A/3L activity [64]. Among the tested 3-halo-3-nitroflavanones, derivatives **47a** and **47b** were identified as leads with IC_50_ values of 0.48 and 0.37 µM, respectively. These findings established a foundation for subsequent structure-based optimisation and medicinal chemistry strategies focused on enhancing potency, selectivity and pharmacokinetic properties. Pechalrieu et al., for example, synthesised a series of analogous 3-halo-3-nitroflavanones as non-nucleoside DNMT inhibitors [69]. SAR studies demonstrated that the presence of both a nitro group and a halogen atom at the C-3 position of the flavanone scaffold was essential for potent DNMT inhibition. Among the synthesised analogues, the 3-bromo-3-nitroflavanones **47c** and **47d** represented in Figure 15 emerged as the lead compounds exhibiting micromolar DNMT inhibitory activity (IC_50_ = 3.2 and 4.7 µM, respectively) with enhanced cytotoxicity against KG-1 leukaemia cells (IC_50_ = 13.0 and 18.6 µM, respectively). Compound **47c** also exhibited moderate cytotoxicity against the HCT116 cell with an IC_50_ value of 10.7 µM). In this case, brominated derivatives consistently outperformed the corresponding chlorinated analogues, suggesting that the larger size and greater polarizability of bromine facilitate stronger interactions within the enzyme active site. These findings established 3-halo-3-nitroflavanones as a promising flavanone-based chemotype for epigenetic therapy and provided valuable guidance for further medicinal chemistry optimisation. The development of these flavanone derivatives complements broader efforts to identify structurally diverse non-nucleoside C5-DNMT inhibitors. Collectively, these studies highlight the evolution of DNMT inhibitor discovery from naturally occurring dietary flavones.

The above results show that strategic functionalisation of the flavone nucleus could generate derivatives with enhanced potency, improved selectivity, and the ability to modulate a diverse range of molecular targets involved in tumour growth, drug resistance, angiogenesis, and immune evasion. Beyond epigenetic modulation, the structural versatility of the flavone scaffold has also enabled its incorporation into hybrid molecules designed to engage multiple oncogenic targets simultaneously. Hybridisation of the flavone scaffold with complementary pharmacophores has therefore been widely employed to enhance antiproliferative potency, improve target selectivity, overcome multidrug resistance, and generate multifunctional molecules with superior therapeutic profiles. Flavone-containing hybrids have emerged as an attractive strategy for the development of anticancer agents and some of the examples are discussed in the next section.

### 2.2. Flavone Hybrids as Anticancer Agents

**Figure 16:**
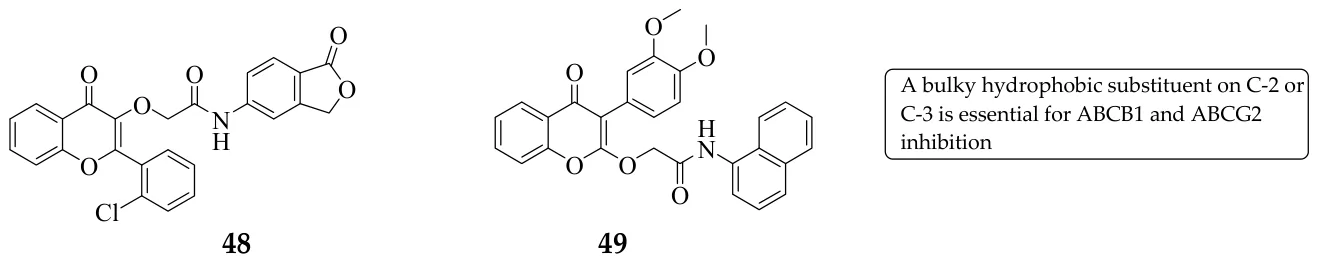
Structures of flavone derivatives **48** and **49**.

More recently, studies have illustrated how rational structural modifications of flavones into molecular hybrids can successfully target diverse cancer-associated proteins, including ATP-binding cassette (ABC) transporters, protein kinases, and histone deacetylases (HDACs). One of the examples of this strategy was reported by Silbermann et al., who designed a series of chalcone-appended flavones and the 3-acetamidoflavone derivatives as selective or dual inhibitors of the multidrug-resistance transporters ABCB1 (P-glycoprotein) and ABCG2 (BCRP) [70]. The aim was to develop selective or dual inhibitors of ATP-binding cassette subfamily B member-1/2 (ABCB1 and (ABCG2) enzymes. Overexpression of ATP-binding cassette (ABC) transporters, particularly ABCB1 (P-glycoprotein) and ABCG2 (BCRP) are associated with multidrug resistance (MDR), a major obstacle in cancer chemotherapy [71,72]. These efflux pumps actively export anticancer drugs from tumour cells, reducing intracellular drug concentrations and therapeutic efficacy. Among the synthesised flavone-based molecular hybrids, compounds **48** and **49** represented in Figure 16 emerged as promising dual modulators of the MDR transporters ABCB1 and ABCG2. The isobenzofuranone-substituted flavone **48** exhibited moderate inhibition against the ABCB1 and ABCG2 transporters with the IC_50_ values of 5.01 μM and 2.90 μM, respectively. In addition, this compound sensitised the resistant cell lines A2780/ADR and MDCK II BCRP with EC_50_ values of 9.90 μM and 1.22 μM, respectively, indicating its ability to reverse transporter-mediated drug resistance. An even more potent profile was observed for the naphthyl-substituted flavone **49**, which inhibited ABCB1 and ABCG2 with the IC_50_ values of 5.43 μM and 3.75 μM, respectively. More importantly, compound **49** effectively restored drug sensitivity in resistant cells, displaying EC_50_ values of 3.12 μM in A2780/ADR cells and 0.446 μM in MDCK II BCRP cells. Its MDR-reversal potency was comparable to that of established dual ABCB1/ABCG2 inhibitors such as WHI-P154 and linsitinib. These findings suggest that incorporation of bulky hydrophobic substituents, particularly naphthyl and isobenzofuranone moieties, enhances interactions with ABC transporters and promotes MDR-reversal activity.

**Figure 17:**
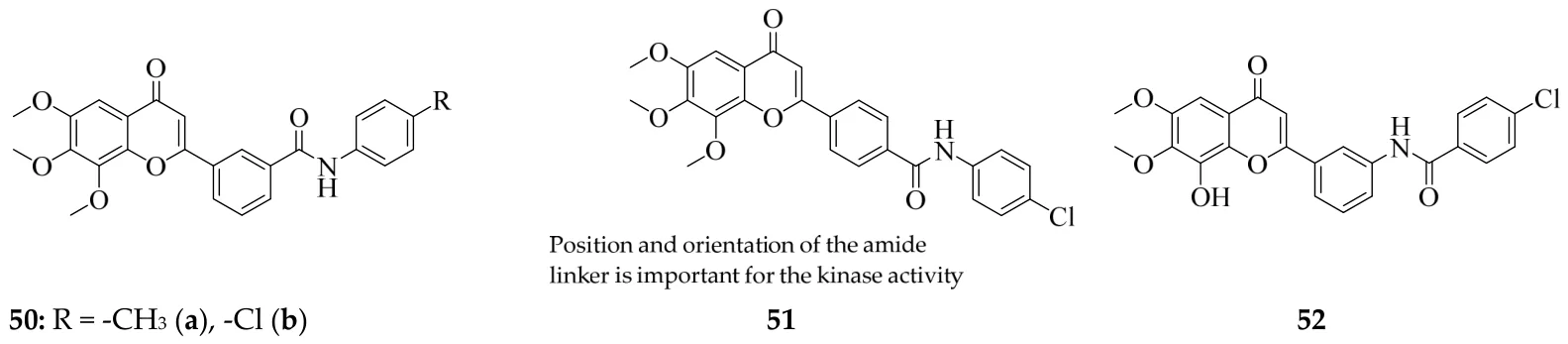
Structures of flavone-based arylamide hybrids **50**, **51** and **52**.

Hassan et al. developed a series of flavone-based arylamide hybrids by combining the flavone scaffold with biologically active arylamide pharmacophores to improve anticancer activity [73]. Biological evaluation using the NCI-60 human tumour cell line panel, kinase profiling, and mechanistic studies identified compounds **50a** and **50b** shown in Figure 17 as the most potent analogues. Both compounds exhibited broad-spectrum antiproliferative activity against panel of the test cancer cell lines in all cases outperforming the clinically used tyrosine kinase inhibitors imatinib and masitinib. They demonstrated submicromolar to low micromolar GI_50_ values against multiple cancer cell lines, with the highest sensitivity observed in colon (COLO205), melanoma (LOXIMVI), renal (CAKI1), lung (HOP92), brain (SNB75), and breast (BT549) cancers (see Table 6). Among these, compound **50a** was selected as the lead candidate because of its superior potency, broader activity spectrum, and favourable selectivity profile. The 5,6,7-trimethoxyflavone scaffold was consistently more active on haematologic, colorectal, central nervous system, ovarian, renal, and breast cancer cell lines compared to the corresponding 5-hydroxy analogues, indicating that increased hydrophobicity enhances cellular potency. Electron-withdrawing halogens on the terminal aryl ring significantly improved antiproliferative activity. Hybrids in which the amide linker is attached to the para position, or where the amide nitrogen is connected to the meta/para position of the flavone B-ring such as in compounds **51** and **52**, respectively, as shown in Figure 17 (GI_50_ values not determined) proved to be less cytotoxic than the analogues with the opposite amide linker configuration. In addition, the orientation and attachment position (3′ or 4′) of the amide bond on the flavone B-ring were critical for optimal activity. Mechanistic studies revealed that compound **50a** induced G_1_-phase cell-cycle arrest in HT29 colon cancer cells and inhibited members of the STE20/GCK-IV kinase family, including HGK (MAP4K4), TNIK, and MINK1. These findings establish compound **50a** as a promising lead for further development of flavone-based kinase-targeted anticancer agents.

**Figure 18:**
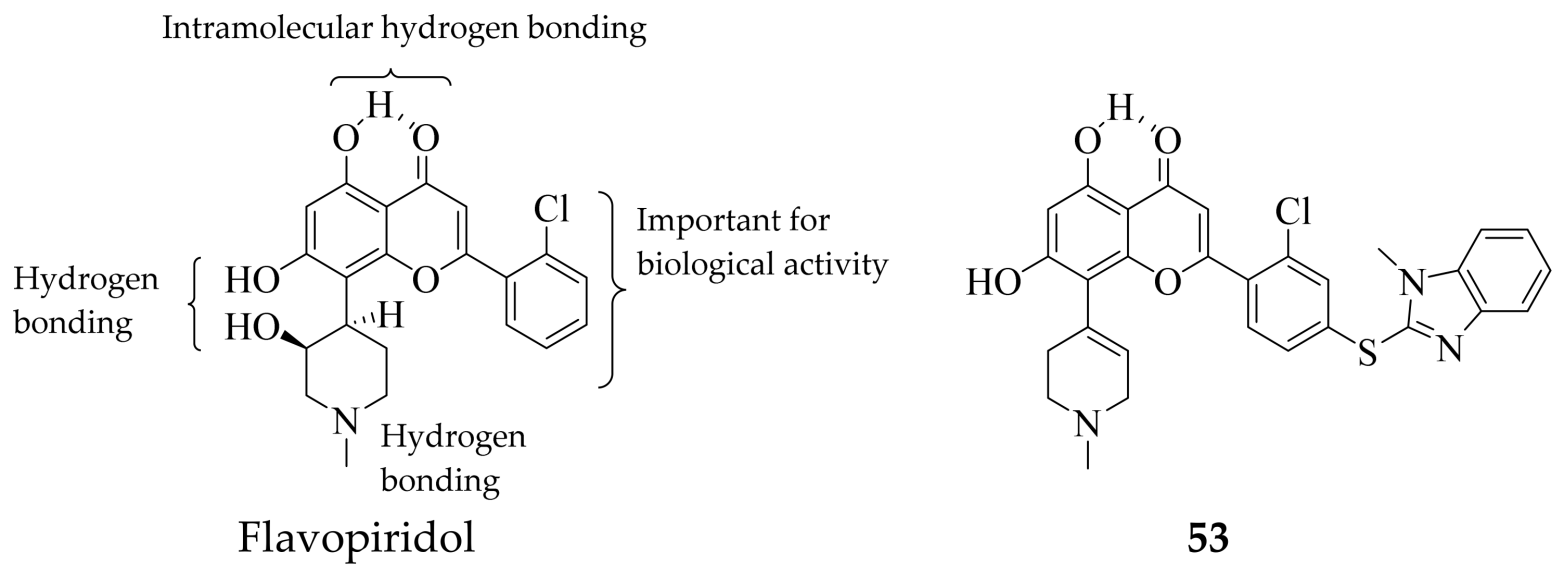
Chemical structures of flavopiridol showing hydrogen-bonding sites, and compound **53**.

Flavopiridol (alvocidib), shown in Figure 18, is a synthetic flavonoid derived from rohitukine with significant anticancer properties primarily driven by its inhibition of CDKs, induction of apoptosis, promotion of oxidative stress, and antiangiogenic activity across various cancer types [74]. The anticancer activity of flavopiridol is attributed to the presence of a chromone moiety that is bioisosteric with the purine ring of adenosine triphosphate (ATP) and binds competitively to the ATP-binding pocket of CDK. The phenyl ring (ring B) interacts with different regions than those occupied by ATP participating in van der Waals-type interactions with other amino acid units thus providing additional contact with the enzyme [75]. The kinase inhibitory activity is due to the presence of hydroxyl groups at C-5 and C-7 positions and eliminating the chlorine atom renders the compound inactive [70]. The hydroxyl groups at C-7 and C-5 positions and the carbonyl group as well as the nitrogen atom and the hydroxyl group of the piperidine moiety of flavopiridol are involved hydrogen-bonding interactions with CDK enhancing the inhibitory effect of this compound against the kinase activity. Despite its encouraging effects, toxicity concerns and the development of drug resistance hampered the clinical application of flavopiridol. Within that context, several investigations were conducted to prepare derivatives of flavopiridol to optimise its anticancer potential against various cancer cell lines [74]. For example, a flavopiridol hybrid **53** (Figure 18) was identified through SAR and this compound was found to have in vitro antiproliferative activity against up to seven cancer cell lines (Table 7) [76]. In addition, compound **53** exhibited strong inhibition against CDK9, CDK10, and GSK3β protein kinases. Although **53** exhibited weaker biochemical inhibition of CDK9 than flavopiridol (IC_50_ = 64 and 20 nM, respectively), it displayed greater overall cytotoxic potency across a panel of cancer cell lines. Notably, compound **53** exhibited a broader kinase profile encompassing CDK9, CDK10 and GSK3β, with nanomolar inhibition of CDK9 and GSK3β, suggesting that its enhanced cellular activity may arise from polypharmacological kinase inhibition rather than increased CDK9 potency alone.

**Figure 19:**
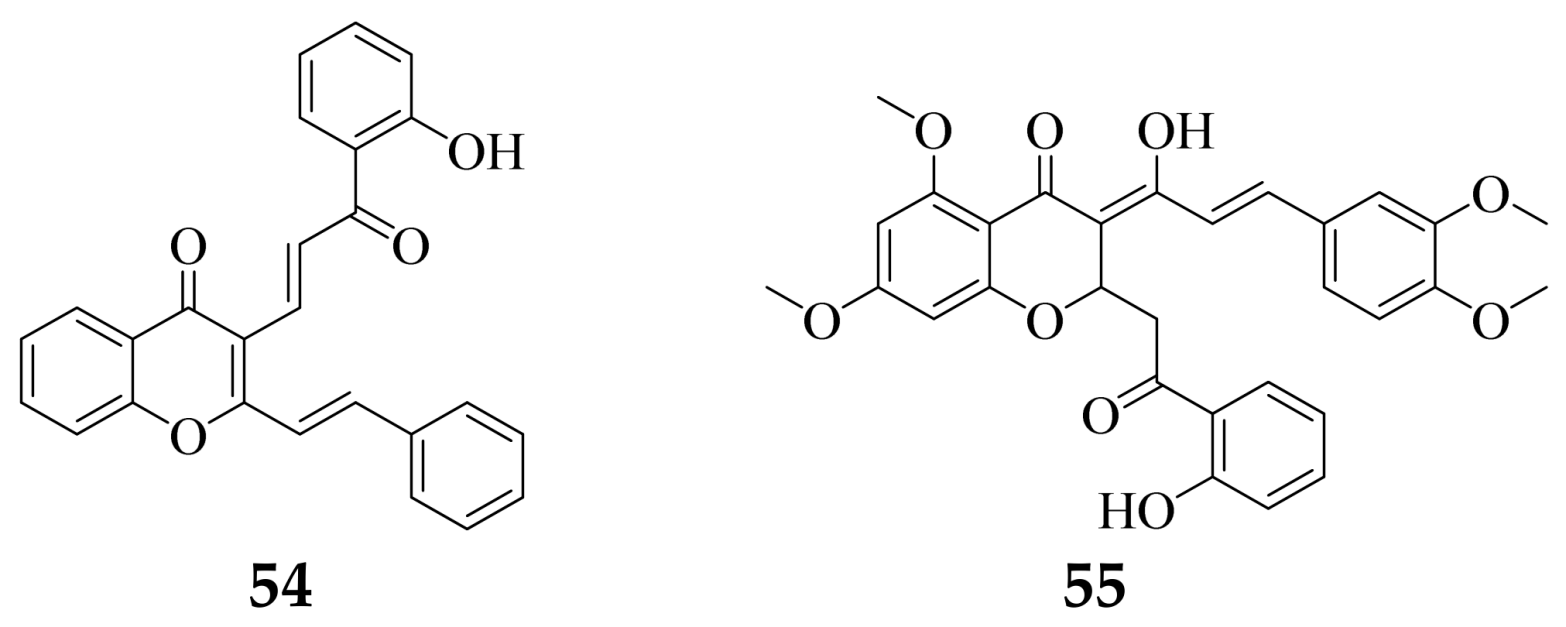
Chemical structures of 2-styrylchromone **54** and chromanone derivative **55**.

Modification of the benzopyran scaffold of flavone-related compounds has also generated derivatives with combined antioxidant and antiproliferative activities. Talhi et al. reported a one-pot synthesis of a library of benzopyran-4-one derivatives, 2-styrylchromones and their chromanone derivatives for further studies of biological activity as anticancer agents [77]. Several compounds exhibited promising antioxidant and antiproliferative activities. Among the test compounds, the 2-styrylchromone derivative **54** and chromanone derivative **55** represented in Figure 19 emerged as the most potent antiproliferative agents against the human K562 leukaemia cells with the IC_50_ values of 4.5 and 7.9 μM, respectively. Notably, these compounds exhibited minimal toxicity towards normal peripheral blood mononuclear cells, suggesting a favourable therapeutic window. SAR analysis revealed several important trends. First, incorporation of an extended styryl moiety at C-2 of the chromone nucleus, as exemplified by compound **54**, markedly enhanced antiproliferative activity, likely due to increased conjugation and improved interactions with cellular targets. Second, the presence of *ortho*-hydroxyl substituents in the aryl ring promoted cyclisation to chromanones and significantly improved both antioxidant and cytoprotective properties.

**Figure 20:**
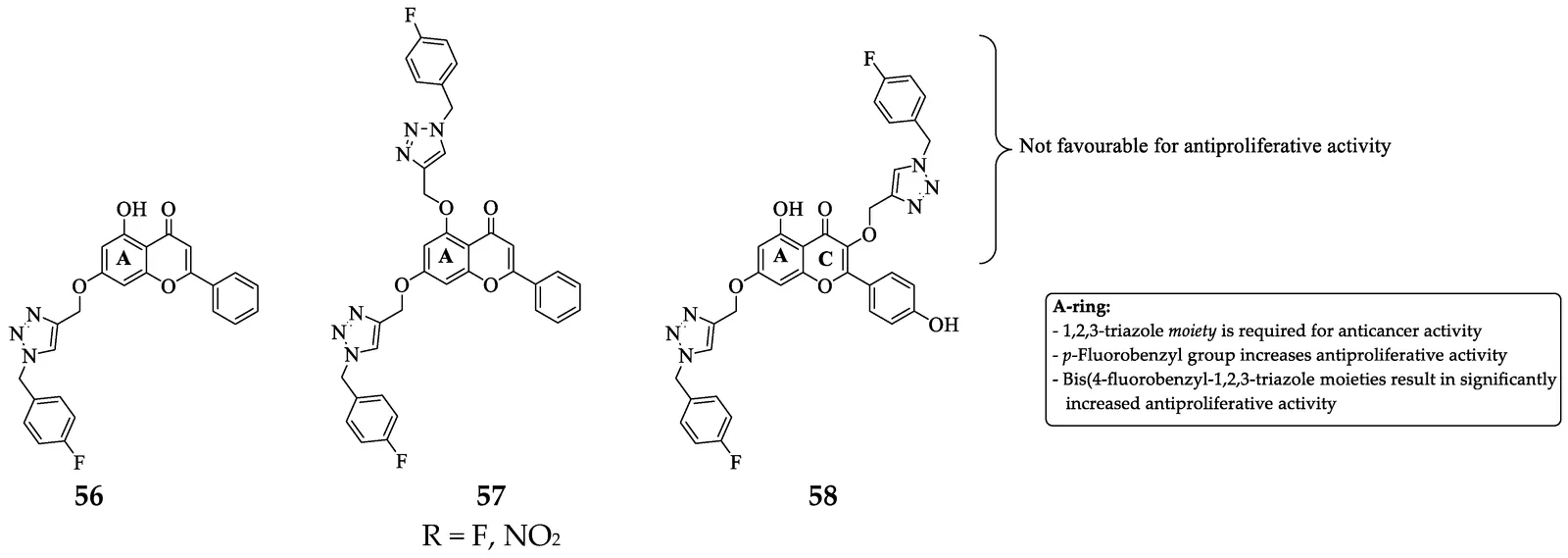
Chemical structures of chrysin hybrids containing one (**56**) or two 1,2,3-triazole units (**57** and **58**).

The hybridisation of the basic skeleton of flavones with other pharmacophore moieties has led to new compounds with superior pharmacological profiles compared to their natural analogues. The 4-fluoro- and 4-nitrobenzyl-1,2,3-triazole-containing chrysin hybrids were prepared and evaluated for cytotoxicity against 60 cell lines of common cancer types (NCI, USA) in vitro [78]. Amongst the tested hybrids, **56** and **57** (Figure 20) where 1,2,3-triazole groups are linked to the A-ring of the flavone through alkoxy linker showed high antiproliferative activity on several cancer cell lines. Notably, both compounds exhibited activity against HOP-62 non-small cell lung cancer cells (GI_50_ = 3.76 and 2.33 µM, respectively), HCT-116 colon cancer cells (GI_50_ = 5.91 µM and 3.52 µM, respectively), and SNB-75 CNS cancer cells (GI_50_ = 3.74 and 1.69 µM, respectively), among other tested cell lines. Interestingly, shifting alkoxy-1,2,3-triazole group from C5 to C3 on C-ring to give hybrid **58** led to modest anticancer activities. Although the list of compounds tested was not exhaustive enough and structural diversity relatively limited, SAR studies revealed that (i) the bis-hybrid compounds exert considerable antiproliferative effect and (ii) replacement of the fluorine atom by a nitro group reduces the bioactivity.

**Figure 21:**
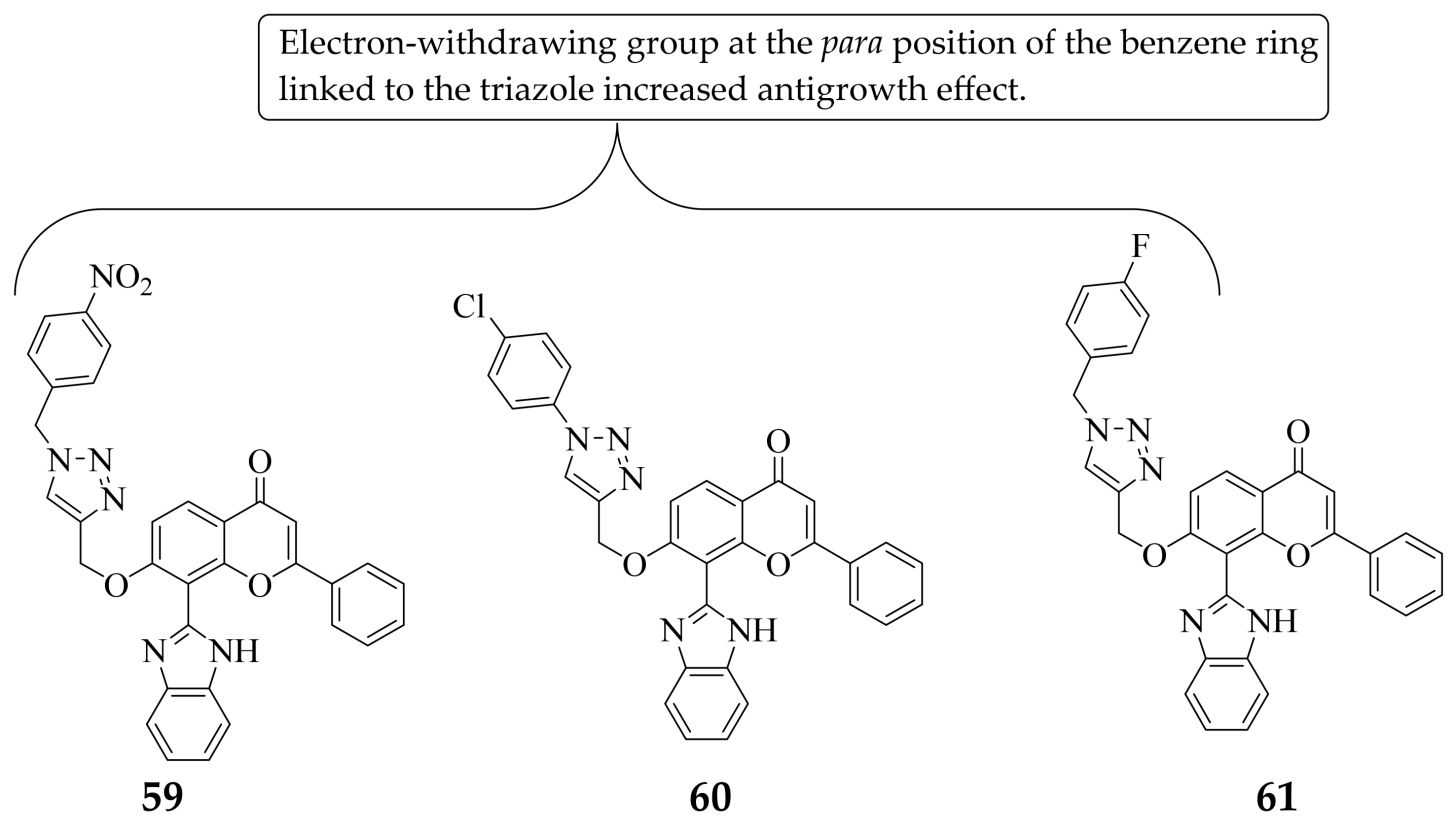
Chemical structures of flavone–triazole–benzimidazole hybrids **59**–**61**.

Flavone/isoxazole-annulated heterocycles and flavone/1,2,3-triazole/benzimidazole hybrids in which the annulated heterocycles are attached to the A-ring of the flavone core were synthesised and evaluated for their antiproliferative activity against the MCF-7 cell line and for antimycobacterial activity [79]. Biological screening using the sulforhodamine B (SRB) assay revealed that most of the flavone/1,2,3-triazole/benzimidazole hybrids exhibited significant antiproliferative activity, highlighting the utility of combining flavone, triazole and benzimidazole pharmacophores within a single molecular framework. In contrast, the flavone/isoxazole-fused derivatives generally exhibited weaker cytotoxic activity, suggesting that annulation of the isoxazole ring alone is insufficient to achieve potent anticancer effects. The presence of a (benzyl-1,2,3-triazole)methoxy group at the 7-position of the A-ring of the flavone–benzimidazole hybrids **59**, **60** and **61** (see Figure 21) resulted in significant cytotoxicity against the MCF-7 cells albeit moderate compared to doxorubicin (IC_50_ = 6.2 µM) with the IC_50_ values of 14.2 μM, 17.9 μM and 19.1 μM, respectively. The presence of an electron-withdrawing group at the *para* position of the benzene ring (e.g., F, Cl or -NO_2_) linked to the triazole moiety further improved the anticancer activity. SAR studies revealed that the position and orientation of substituents on the 1,2,3-triazole ring and on the flavone–benzimidazole framework play a critical role in determining antiproliferative activity against the MCF-7 cancer cell line. Besides anticancer evaluation, several compounds also demonstrated antimycobacterial activity, although only moderate inhibition was observed.

**Figure 22:**
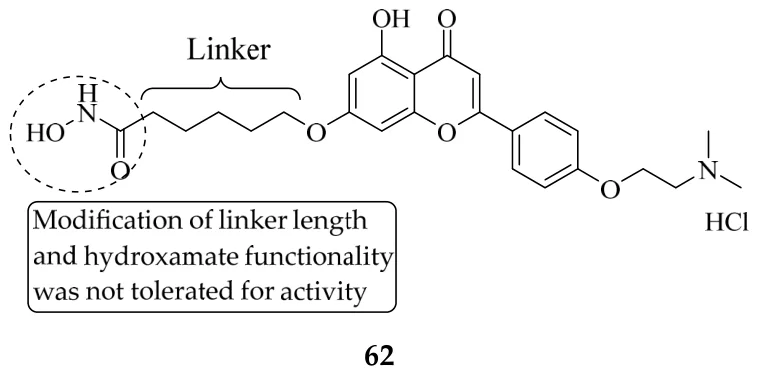
Chemical structure of lead compound **62**.

A series of flavone- and isoflavone-based histone deacetylase (HDAC) inhibitors was synthesised by integrating flavonoid scaffolds as hydrophobic cap groups into the classical hydroxamic acid pharmacophore [80]. The hydroxamic group is a preferred zinc-binding moiety in the design of new HDAC inhibitors and its advantages include superior zinc-binding ability, fair in vitro stability, good solubility, and easy synthesis [81]. This rational hybridisation strategy was aimed at improving the limited efficacy of conventional HDAC inhibitors against solid tumours while simultaneously harnessing the inherent anticancer properties of flavones. In vitro biological activity assays identified compound **62** (Figure 22) as the lead candidate, demonstrating potent antiproliferative activity across a broad panel of human cancer cell lines, including breast, melanoma, hepatocellular, lung, and pancreatic cancers. Compound **62** exhibited greater antiproliferative potency than suberoylanilide hydroxamic acid (SAHA) against most of the cancer cell lines evaluated (see IC_50_ values against selected cell lines in Table 8). This compound exhibited pronounced activity against SAHA-resistant breast cancer cells. Compound **62** selectively inhibited HDAC1, HDAC2, HDAC3 and HDAC6, resulting in increased acetylation of histones and α-tubulin, induction of G1/S cell-cycle arrest, and apoptosis. Unlike many HDAC inhibitors that activate the pro-survival STAT3 pathway, compound **62** also suppressed STAT3 activation. This compound reduced the expression of STAT3-regulated survival proteins and enhanced antitumour efficacy both in vitro and in vivo. SAR studies demonstrated that the flavone scaffold served as an effective cap group for HDAC inhibition. Incorporation of a hydroxamic acid zinc-binding group through an appropriately sized linker was found to be essential for potent activity. Flavone-based derivatives consistently outperformed the corresponding isoflavone analogues, indicating that the position and orientation of the heterocyclic core play a critical role in target engagement. The optimised linker length and hydroxamate functionality enabled efficient interaction with the HDAC catalytic zinc ion. Modifications to these features markedly reduced antiproliferative activity, highlighting the importance of maintaining the classical HDAC pharmacophore within the flavone framework.

Collectively, these studies demonstrate that hybridisation represents one of the most successful strategies for optimising the anticancer potential of flavones. Depending on the pharmacophore introduced, flavone hybrids can effectively target multidrug-resistance transporters, protein kinases, HDACs, and oxidative stress pathways.

### 2.3. In Silico Approaches for the Design of Flavones as Anticancer Agents

Computer-aided drug design (CADD) has become an indispensable tool for elucidating ligand–target interactions, predicting biological activity, and guiding the design of more potent flavone-based therapeutics. This strategy compliments experimental approaches in both synthetic and biological studies by enabling prioritisation of structurally promising leads. Commonly used approaches include pharmacophore mapping, molecular docking, quantitative structure–activity relationship (QSAR) modelling, virtual screening, and ADME/Tox prediction, with each method providing complementary information [82,83]. In this context, Simon et al. in their study employed an integrated in silico-guided screening strategy to identify flavone-based leads with potential activity against colon cancer [84]. The study demonstrated how pharmacophore mapping, molecular docking, and Monte Carlo-based QSAR modelling can accelerate the discovery and optimisation of antiproliferative flavones against the human colon cancer HCT116 cell line. Inverse pharmacophore screening against several cancer-related proteins identified cyclin-dependent kinase-2 (CDK-2) as the most relevant target associated with the observed antiproliferative activity. Pharmacophore mapping identifies the essential steric, hydrophobic, aromatic, and hydrogen-bonding features required for biological recognition and can therefore rapidly screen structurally diverse compounds. This approach is particularly useful for hit identification and scaffold hopping because compounds with different chemical backbones can be identified if they reproduce the required pharmacophoric features [85]. The models of this approach, however, are highly dependent on the quality and variety of the compounds used to generate them and may not adequately account for conformational changes, receptor flexibility, solvent effects, or alternative binding modes. Consequently, a pharmacophore match indicates potential activity but does not establish biological efficacy and requires experimental validation [85,86]. Subsequent molecular docking confirmed favourable interactions between selected flavonoids and the ATP-binding pocket of CDK-2, supporting its role as a key mediator of growth inhibition consistent with the in vitro results previously observed for flavopiridol [76]. Molecular docking provides a three-dimensional prediction of ligand orientation within a protein binding site and allows specific hydrogen-bonding, hydrophobic, electrostatic and π–π interactions to be examined. This information is valuable for rational flavone optimisation because structural modifications can be related to predicted changes in target recognition before compounds are synthesised [75]. However, protein structures in molecular docking are generally treated as relatively rigid, whereas proteins undergo conformational changes during ligand binding. Furthermore, scoring functions provide approximations of binding affinity and may not accurately account for solvent effects, receptor flexibility or all components of the binding free energy. Therefore, a favourable docking score should be interpreted as a hypothesis of potential binding rather than definitive evidence of biological activity [87].

**Figure 23:**
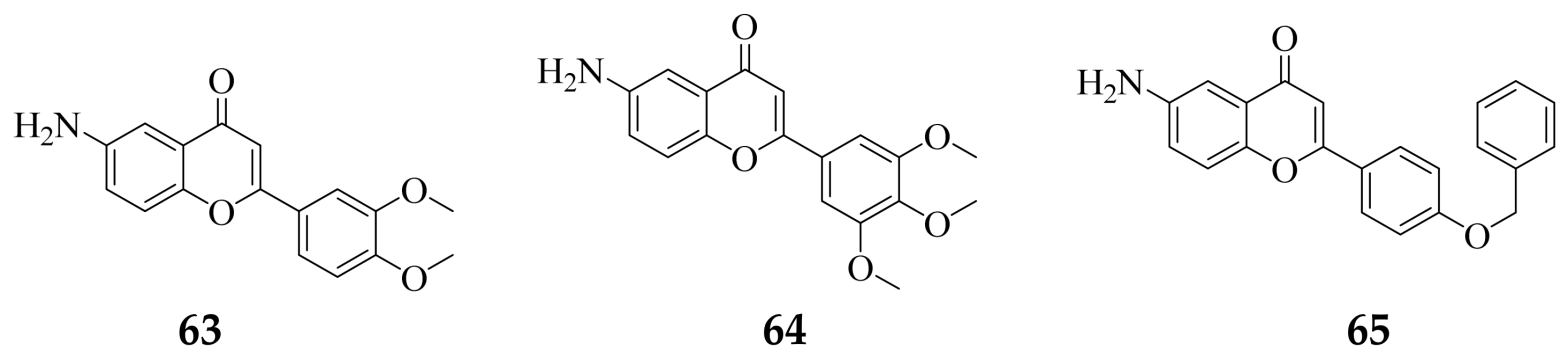
Chemical structures of the flavone derivatives **63**–**65**.

Monte Carlo-based QSAR analysis of a series of substituted flavones revealed that B-ring substitution strongly influenced antiproliferative potency. The most promising leads included compounds **63**, **64**, and **65** (Figure 23). Compound **63** displayed the highest activity against HCT116 cells compared to **64** and **65** with the pIC_50_ values of 4.6, 4.52 and 4.53, respectively. These compounds also showed the strongest molecular docking scores, particularly **65** (MolDoc score = −124.750), suggesting enhanced affinity for CDK-2. The SAR analysis indicated that electron-donating substituent/s on the B-ring, especially the methoxy and benzyloxy groups, significantly enhanced anticolon cancer activity. In contrast, unsubstituted flavones or derivatives bearing only single hydroxyl groups displayed reduced potency. The presence of multiple methoxy groups improved hydrophobic interactions within the kinase binding pocket, whereas bulky benzyloxy substitution further strengthened ligand-protein interactions. Monte Carlo-based QSAR modelling corroborated these findings by identifying hydrophobic fragments and specific hydrogen-bonding features as positive contributors to activity. Monte Carlo-based QSAR modelling can establish quantitative relationships between molecular structure and biological activity and predict the activity of analogues before their synthesis. It can identify structural fragments that positively or negatively influence potency, thereby providing a rational basis for selecting compounds for synthesis and reducing experimental workload [88]. However, QSAR models are strongly dependent on the quality, size and chemical diversity of the training dataset. They may also suffer from overfitting and generally provide reliable predictions only for compounds falling within their applicability domain. Thus, predictions for structurally distinct flavones may be unreliable, and statistical correlation should not be interpreted as definitive evidence of a particular biological mechanism [82,88]. Overall, the study established methoxy-rich and benzyloxy-substituted flavones as promising lead scaffolds for the development of novel anticolon cancer agents targeting CDK-2 inhibition.

Computational approaches have also been applied to identify flavone derivatives targeting cancer-associated signalling pathways. Tankyrase (TNKS), a member of the poly(ADP-ribose) polymerase (PARP) family, is a key regulator of the Wnt/β-catenin signalling pathway through the poly(ADP-ribosyl)ation and subsequent degradation of AXIN, a critical component of the β-catenin destruction complex. Aberrant TNKS activity promotes sustained Wnt/β-catenin signalling, a hallmark of many human cancers, making TNKS an attractive target for anticancer drug discovery [89]. Owing to their structural diversity and favourable pharmacological properties, flavones and their analogous flavanones have attracted considerable attention as promising TNKS inhibitors capable of suppressing Wnt/β-catenin signalling. Using an integrated computer-aided drug design (CADD) approach, Alam and Khan combined 3D-QSAR, molecular docking, virtual screening, and ADME/Tox prediction to identify novel flavone analogues with enhanced tankyrase inhibitory and anticancer potential [90]. The field point-based 3D-QSAR model exhibited strong predictive capability (r^2^ = 0.89, q^2^ = 0.67), demonstrating its reliability in correlating structural features with biological activity and guiding the rational design and optimisation of flavone-based TNKS inhibitors. The SAR analysis revealed that the electrostatic, steric, and hydrophobic characteristics surrounding the flavone scaffold play critical roles in tankyrase inhibition. Substituents capable of enhancing favourable hydrogen-bonding interactions while maintaining appropriate hydrophobic contacts within the tankyrase binding pocket significantly improved predicted potency. Virtual screening of approximately 8000 flavonoids identified 1480 compounds with predicted IC_50_ values below 5 μM, which were subsequently prioritised using molecular docking and pharmacokinetic filtering. Virtual screening can computationally analyse thousands to millions of compounds before hands-on experiments. This can greatly reduce the chemical space that must be investigated experimentally, lowering the cost and time associated with early-stage drug discovery [91]. One of the drawbacks of virtual screening is that it is susceptible to both false-positive and false-negative predictions because it relies on the quality of the screening library, molecular conformations, pharmacophore assumptions, docking protocols and scoring functions. Consequently, computationally prioritised compounds should be regarded as candidates for experimental testing rather than confirmed active compounds [92].

**Figure 24:**
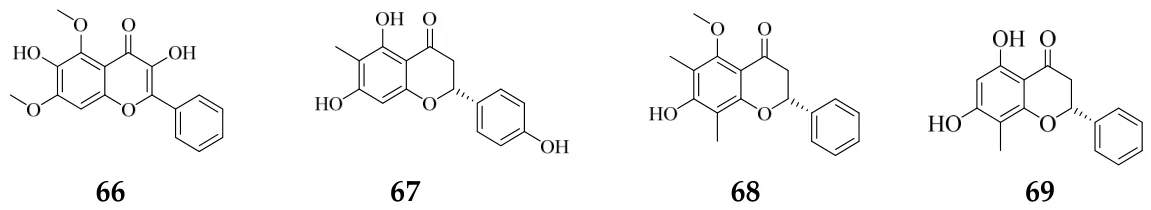
Chemical structures of flavone **66** and flavanone derivatives **67**–**69**.

Following ADME/Tox evaluation, only 25 compounds satisfied drug-likeness, oral bioavailability, synthetic accessibility, and PAINS/Brenk filtering criteria. Among these, flavonol **66** and flavanones **67**–**69** (Figure 24) emerged as the most promising leads, with predicted IC_50_ values of 0.62, 0.63, 0.64, and 0.79 μM, respectively. Docking studies demonstrated favourable interactions within the TNKS2 catalytic pocket, supporting their proposed mechanism of action. Furthermore, ADME/Tox analyses predicted acceptable pharmacokinetic properties, low toxicity risk, and favourable oral bioavailability, highlighting these compounds as promising candidates for further synthesis and biological validation. The ADME/Tox prediction tools provide advanced consideration of pharmacokinetic and toxicity liabilities actual prioritised compounds can be synthesised and tested [93]. However, these predictions remain model-dependent approximations and cannot fully reproduce the complexity of human pharmacokinetics or toxicity. As a result, predicted favourable ADME/Tox properties should not be interpreted as evidence of human safety or efficacy and require experimental confirmation. Collectively, this study illustrates the value of integrating QSAR, molecular docking, and ADME/Tox profiling to accelerate the identification and optimisation of flavone-derived tankyrase inhibitors as potential anticancer agents.

**Figure 25:**
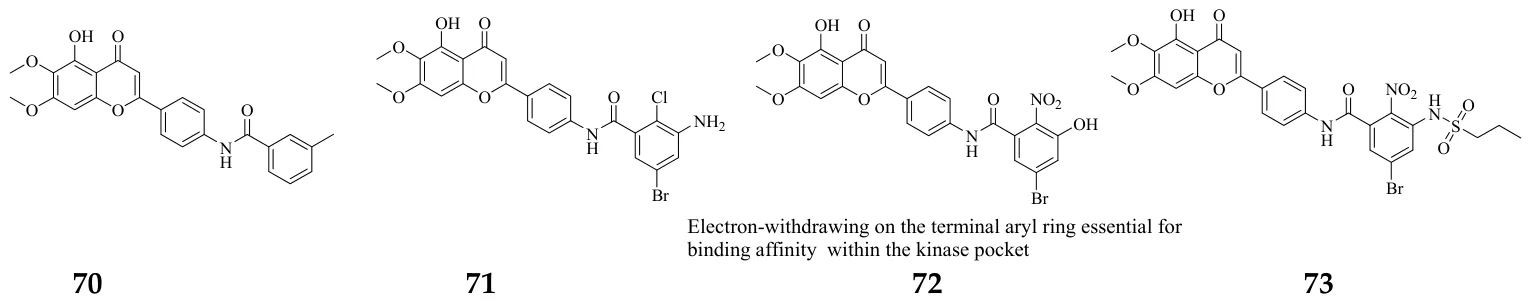
Chemical structures of flavone-based arylamide analogues **70**–**73** designed through structure-based optimisation for V600E-BRAF inhibition.

Computational methodologies have likewise facilitated the rational design of flavone derivatives targeting mutant protein kinases involved in oncogenesis. A docking strategy was employed to develop flavone-derived inhibitors of the mutant V600E-BRAF kinase, a key oncogenic target implicated in melanoma [94]. The designed library of flavone-based arylamide analogues was evaluated theoretically using molecular docking in V600E-BRAF (PDB ID: 3OG7) and their drug-likeness and ADMET (Absorption, Distribution, Metabolism, Excretion, and Toxicity) were predicted to identify promising anticancer leads. Within the tested dataset, compound **70** (Figure 25) exhibited stronger predicted binding affinity (molecular docking score = −160.581) toward the V600E-BRAF active site than the reference inhibitor vemurafenib (molecular docking score = −158.139). Compound **70** was, in turn, used as a template to design derivatives **71**–**73** (Figure 25) which showed efficient predicted binding within the active sites of the target as shown in Table 9. The binding affinities of compounds **71** and **73** outperformed that of the reference drug. These compounds established multiple hydrogen-bonding and hydrophobic interactions with critical amino acid residues within the kinase binding pocket, suggesting favourable inhibition of BRAF signalling. The SAR analysis demonstrated that biological activity was strongly influenced by the nature and position of substituents on both the flavone scaffold and the arylamide moiety. The flavone nucleus served as an essential pharmacophore for anchoring the molecule within the ATP-binding pocket of V600E-BRAF. Introduction of amide linkages, on the other hand, improved molecular orientation and facilitated hydrogen bond interactions with catalytic residues. Electron-withdrawing substituents on the terminal aryl ring generally enhanced binding affinity by strengthening hydrophobic and electrostatic interactions within the kinase pocket. In addition, increased aromatic surface area improved π–π stacking interactions with hydrophobic residues lining the active site. The lead compounds satisfied Lipinski’s rule of five, indicating favourable oral drug-likeness. Predicted ADMET profiles suggested acceptable intestinal absorption, reasonable bioavailability, supporting their suitability for synthesis, further optimisation and biological evaluation.

## 3. Future Perspectives and Emerging Strategies in Flavone-Based Anticancer Drug Discovery

**Figure 26:**
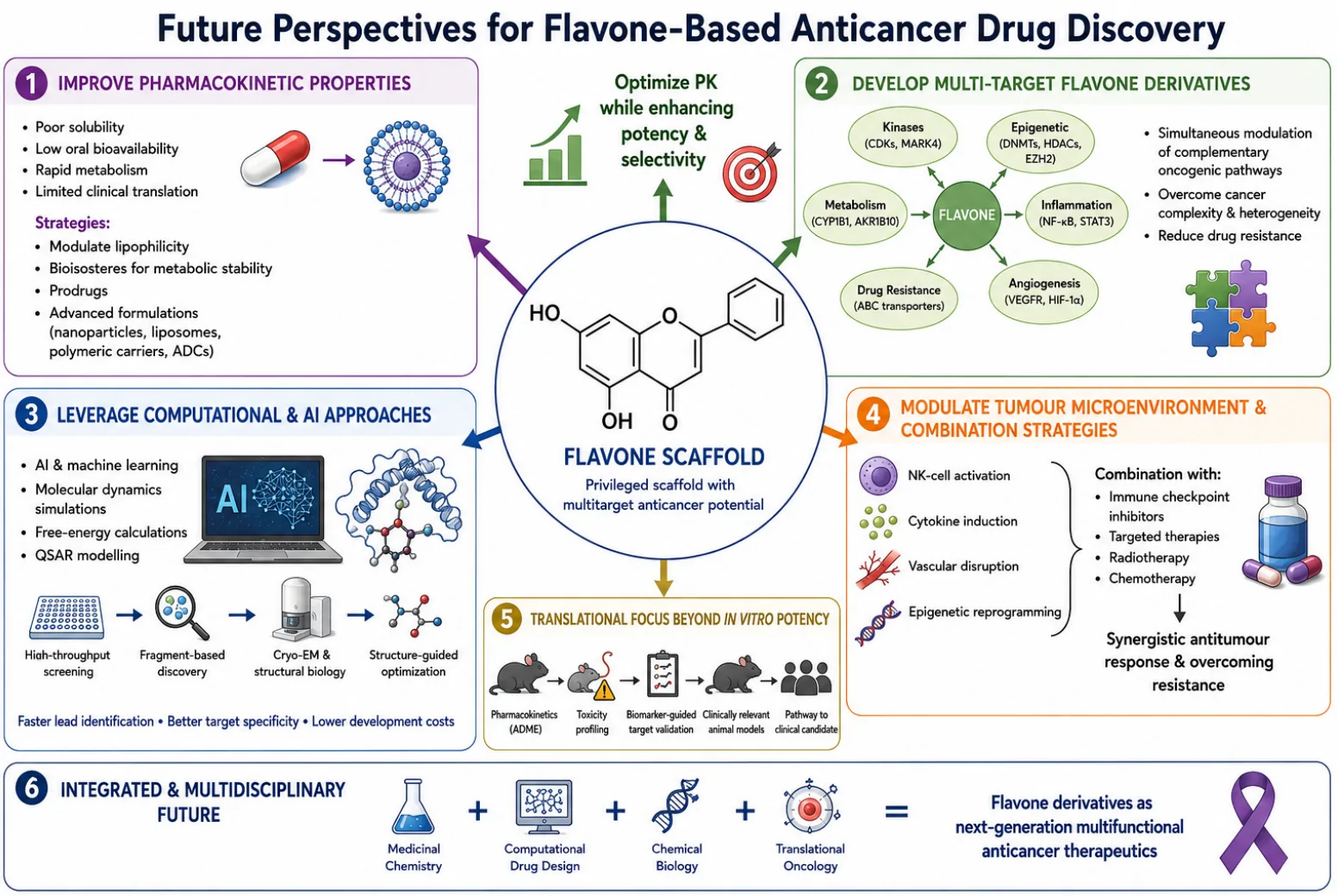
Future perspectives and emerging strategies for flavone-based anticancer drug discovery. Image generated by the authors.

Despite significant advances in flavone-based anticancer drug discovery, few candidates have progressed to clinical development because of poor aqueous solubility, limited oral bioavailability, rapid metabolic conjugation, and an incomplete understanding of their mechanisms of action [26]. As depicted in Figure 26, future research should focus on improving pharmacokinetic properties while maintaining high potency and selectivity through rational structural modification, metabolically stable bioisosteres, prodrug design, and advanced drug delivery systems. The development of multifunctional flavone derivatives capable of simultaneously targeting kinases, epigenetic enzymes, inflammatory pathways, angiogenesis, and drug resistance mechanisms represent a promising strategy to address tumour heterogeneity and overcome resistance [95,96]. Computational approaches, including artificial intelligence (AI), machine learning, molecular dynamics simulations, and quantitative structure–activity relationship (QSAR) modelling, integrated with structure-based drug design and high-throughput screening, are expected to accelerate lead identification while improving ADMET properties [97,98]. The investigations should also elucidate the influence of flavones on the tumour microenvironment, particularly their ability to regulate immune responses through natural killer cell activation, cytokine modulation, vascular disruption, and epigenetic reprogramming, while evaluating synergistic combinations with immune checkpoint inhibitors, targeted therapies, radiotherapy, and conventional chemotherapy [98]. Finally, comprehensive pharmacokinetic, toxicity, and biomarker-guided studies using clinically relevant animal models are essential to establish robust relationships between chemical structure, target engagement, and therapeutic efficacy. The continued integration of medicinal chemistry, computational drug design, chemical biology, and translational oncology is expected to facilitate the development of flavone derivatives and their molecular hybrids as the next-generation multifunctional anticancer therapeutics.

## 4. Conclusions

Flavones have firmly established themselves as privileged scaffolds in anticancer drug discovery because of their ability to modulate multiple molecular targets and signalling pathways involved in tumour growth, metastasis, angiogenesis, immune evasion, and therapeutic resistance. Evidence presented in this review demonstrates that rational SAR studies have been instrumental in identifying key structural features that enhance target affinity, potency, selectivity, and pharmacokinetic properties of flavones. Strategic modifications, including hydroxylation, methoxylation, halogenation, heterocycle incorporation, hybridisation, and bioisosteric replacement, have successfully transformed the naturally occurring flavones into more potent and drug-like anticancer agents with improved target engagement, cellular uptake, metabolic stability, and, in some cases, tumour selectivity. Importantly, the reviewed SAR studies indicate that the biological effects of structural modification are highly target- and context-dependent, with substitution patterns that enhance activity against one target potentially diminishing activity against another. This highlights the importance of target-specific SAR optimisation rather than reliance on a universal substitution pattern. Beyond direct cytotoxicity, flavones exhibit valuable polypharmacological properties by jointly regulating diverse signalling networks and components of the tumour microenvironment, making them attractive candidates for overcoming tumour heterogeneity and drug resistance. The studies reviewed further demonstrate that flavone derivatives can modulate protein kinases, metabolic enzymes, epigenetic regulators, multidrug-resistance transporters, tumour vasculature, inflammatory pathways, and immune-mediated tumour killing. Flavone-based hybrids, on the other hand, provide additional opportunities to engage complementary pharmacological targets. However, the clinical translation of flavone-based therapeutics remains limited by insufficient in vivo validation, pharmacokinetic characterisation, and standardised SAR datasets. Additional challenges include poor aqueous solubility, limited and variable oral bioavailability metabolic instability, and the potential for off-target effects. Furthermore, computational approaches such as molecular docking, QSAR, virtual screening, pharmacophore modelling, and ADME/Tox prediction should be regarded as complementary tools for hypothesis generation and lead prioritisation rather than substitutes for experimental validation. Future research should integrate advanced medicinal chemistry with artificial intelligence-assisted design, structural biology, systems pharmacology, nanotechnology-based delivery and biomarker-driven precision oncology to accelerate lead optimisation and clinical development. Collectively, SAR-guided optimisation positions flavones as a highly promising chemical platform for the development of safer, more selective, and clinically effective multitarget anticancer therapeutics.

## Figures and Tables

**Table 1:** Focused anticancer SAR summary of reviewed flavone derivatives.

Structural Feature/Modification	Representative Compounds	Key SAR Finding/Biological Outcome
Flavone core and phenolic OH	**1**–**6**	Free phenolic OH groups are important for activity. Maintaining the aromatic A- and B-rings supports biological activity.
C=O → C=S bioisosteric replacement	**3**–**6**	Thioflavones showed enhanced antiproliferative potency versus parent flavones; increased sulphur size/polarizability alters physicochemical and binding properties.
A-ring OH position	**3** vs. **4**	5,7-dihydroxy substitution was more favourable than 7,8-dihydroxy substitution for antiproliferative activity.
*para*-Halogenation of B-ring	**5, 6**	*p*-Br/Cl substitution improved cytotoxicity, associated with increased lipophilicity and favourable cellular/target interactions.
B-ring hydroxylation/catechol	**11**–**13**, **19**–**20**	7-OH and B-ring OH groups promote enzyme binding; 3′,4′-dihydroxy substitution is a key pharmacophore for several anticancer/antioxidant activities.
C2=C3 unsaturation/planarity	**7**–**10**, **14**–**18**	The planar flavone framework generally favours antiproliferative and enzyme inhibitory activity compared with saturated flavanones.
Hydroxyl methylation	**17**–**18;** methylated analogues	Methylation of key OH groups generally reduced activity, highlighting the importance of free hydroxyl groups.
Methoxy substitution	**22**–**26**	Activity depends strongly on position and number of OCH_3_ groups; 5-OCH_3_ and balanced A/B-ring methoxy–hydroxy patterns can improve potency and lipophilicity.
C-3 O-alkylation/aminoalkyl chains	**27**–**28**	Converting 3-OH to alkoxyaminoalkyl groups enhanced prostate cancer activity; a three-carbon linker and dibutylamino group were optimal.
8-Aryl/styryl/arylethynyl substitution	**23a**–**e**	Small phenyl/halogenophenyl groups and π-conjugated bridges improved activity against VEGFR-2/EGFR; hydrophobic and H-bond interactions supported binding.
*meta*-EWG on 2-phenyl ring	**29**–**31**	*m*-Cl or m-CF_3_ substitution enhanced CYP1B1 inhibition and selectivity; lead compounds reached sub-nanomolar potency.
EWG on *N*-aryl ring	**32**–**34**	*para*-EWGs improved MARK4 inhibition and antiproliferative activity through favourable ATP-site interactions.
C-6 amino substitution	**41**–**44**	Amino substitution, particularly at C-6, enhanced NK-cell-mediated tumour killing.
B-ring OH for epigenetic targets	**11**, **12** and **45**	B-ring hydroxylation, especially the 3′,4′-dihydroxy pattern, strengthened DNMT/EZH2 inhibition and DNA-binding interactions.
3-Nitro + 3-halo substitution	**46**–**47**	Nitro and halogen groups at C-3 enhanced DNMT inhibition; brominated analogues generally outperformed chlorinated counterparts.
Bulky isobenzofuranone or naphthyl substitution	**48**–**49**	Bulky hydrophobic groups can strengthen ABC–transporter interactions and improve MDR-reversal activity.
Extended styryl group; *ortho*-OH enabling cyclisation to chromanone	**54** and **55**	Extended conjugation can enhance antiproliferative activity; ortho-OH promotes cyclisation and antioxidant/cytoprotective properties.
A-ring alkoxy-1,2,3-triazole; mono- vs. bis-hybrid; F vs. NO_2_	**56**–**58**	Bis-hybridisation favoured; fluorine was preferable to nitro group; attachment position strongly affects activity.
A-ring annulated triazole/benzimidazole framework; para EWG	**59**–**61**	Substituent position/orientation is critical; *para* electron-withdrawing groups on the benzene ring improve activity.

**Table 2:** The IC_50_ values of representative flavones and flavanone against human cancer and normal cell lines.

Compound	IC_50_ Values (µM)						
	MV-4-11	MCF7	LoVo	LoVo/DX	DU 145	MCF 10A	HLMEC
**7**	8.18	22.50	13.80	11.58	28.86	35.67	14.06
**8**	8.73	25.50	13.45	13.69	21.94	33.12	9.22
**9**	ND	ND	235.29	ND	ND	ND	122.21
**10**	ND	121.80	ND	ND	ND	ND	110.22

ND: Not determined in the range of tested concentrations (0.1, 1, 10 and 100 µg/mL).

**Table 3:** Designation of groups Ar and R and the IC_50_ values **23** against VEGFR-2 and EGRF.

			IC_50_ (µM ± SD)		
23	Ar	R	VEGFR-2	EGFR	SI
**23a**	-C_6_H_5_	-C_6_H_4_(4-F)	ND	ND	ND
**23b**	-C_6_H_5_	-CH=CHC_6_H_4_(4-F)	14.41 ± 0.03	5.04 ± 0.30	2.86
**23c**	-C_6_H_4_(4-Cl)	-C_6_H_5_	7.59 ± 0.03	3.87 ± 0.31	1.96
**23d**	-C_6_H_5_	-C≡CC_6_H_5_	ND	ND	ND
**23e**	-C_6_H_5_	-C≡CC_6_H_4_(4-F)	5.72 ± 0.04	8.31 ± 0.33	0.67
Quercetin	-	-	4.30 ± 0.05	27.48 ± 0.14	0.16
Nintendanib	-	-	0.42 ± 0.03	17.65 ± 0.28	0.02

ND: Not determined in the range of tested concentrations (1, 5, 10, 25 and 50 μM).

**Table 4:** IC_50_ values of compounds **35**–**40** against the A549, HT-29 and HPBMCs.

	IC_50_ Values (M)		
Compound	A549	HT-29	HPBMCs
**35**	291 ^a^ & 84 ^b^	239 ^a^	>500 ^a^
**36**	239 ^a^ & 86 ^b^	217 ^a^	416 ^a^
**37**	>500 ^a^ & 93 ^b^	>1000 ^a^	>500 ^a^
**38**	>500 ^a^ & 39 ^b^	>500 ^b^	>1000 ^a^
**30**	>500 ^a^ & 45 ^b^	>500 ^b^	>882 ^a^
**40**	>500 ^a^ & 36 ^b^	>500 ^b^	891 ^a^

^a^ Direct cytotoxicity; ^b^ indirect cytotoxicity.

**Table 5:** Cytotoxicity results (IC_50_ values) of **41**–**44** against the A549, H1975 and MRC5 cells.

	IC_50_ Values (M)		
Compound	A549	H1975	MRC5
**41**	24.20	77.27	109.70
**42**	23.14	38.65	70.12
**43**	56.13	72.78	>100
**44**	34.38	53.84	>100

**Table 6:** GI_50_ (µM) values of **50a** and **50b** against selected NCI-60 human cancer cell lines.

Compound	GI_50_ (µM)								
	RPMI8226	HOP92	COLO205	SNB75	DU145	LOXIMVI	ADRRES	CAKI1	BT549
**50a**	0.37	1.55	1.05	1.16	3.21	0.32	1.46	0.32	1.71
**50b**	0.49	1.46	1.47	2.66	2.85	0.41	1.81	1.79	1.18
Imatinib	6.05	13.34	17.62	20.28	18.79	18.11	22.96	33.96	16.11
Masitinib	3.8	11.48	4.39	14.5	15.56	2.17	23.55	12.45	11.94

**Table 7:** Inhibitory effects on cell viability of compound **53** against a panel of seven human cancer cell lines.

Cell Line	SKOV3	NCI-N87	SKBR3	PC3	MiaPaCa-2	HCT116	K562
IC_50_ (µM)	0.09	0.05	0.05	0.09	0.05	0.18	0.05

**Table 8:** Cytotoxicity (IC_50_ µM) of **62** and SAHA against a panel of human cancer cell lines.

IC_50_ (µM)		
Cell Line	62	SAHA
Sum-159	0.23	1.38
B16F10	0.51	2.07
PLC	0.58	1.53
SMMC7721	0.53	1.99
A375	1.18	2.83
HepG2	1.16	2.27
A549	1.08	2.36

**Table 9:** Docking parameters values for compounds **70**–**73**.

Parameter	70	71	72	73
MolDock score	−160.581	−164.145	−148.244	−166.413
Log P	4.85	4.79	4.65	5.09
TPSA	98.00	124.02	153.05	198.37
Bioavailability	0.55	0.55	0.17	0.17
Synthetic accessibility	3.43	3.60	3.93	4.47
Intestinal absorption (%)	100	84.75	84.49	77.68
HBA/HBD	6/2	6/3	9/2	10/3

MolDock = Molecular docking; TPSA = topological polar surface area; HBA = hydrogen bond acceptor; HDB = hydrogen bond donor.

## Data Availability

No new data were created or analyzed in this study. Data sharing is not applicable to this article.

## Abbreviations

The following abbreviations are used in this manuscript:

SAR	Structure–Activity Relationship
PARP	Poly(ADP-ribose) Polymerase
PI3K	Phosphoinositide 3-Kinase
mTOR	Mechanistic Target of Rapamycin
MAPK	Mitogen-Activated Protein Kinase
ERK	Extracellular Signal-Regulated Kinase
JAK	Janus Kinase
STAT3	Signal Transducer and Activator of Transcription 3
NF-κB	Nuclear Factor Kappa-Light-Chain-Enhancer of Activated B Cells
p53	Tumour Protein p53
HIF-1α	Hypoxia-Inducible Factor 1 Alpha
TGF-β	Transforming Growth Factor Beta
GSK-3β	Glycogen Synthase Kinase-3 beta
HDAC	Histone deacetylase
CDK-2	Cyclin-dependent kinase-2
QSAR	Quantitative Structure–Activity Relationship
ATP	Adenosine Triphosphate
TNKS	Tankyrase
EWG	Electron-Withdrawing Group
